# The Identification of Enteric Fever-Specific Antigens for Population-Based Serosurveillance

**DOI:** 10.1093/infdis/jiad242

**Published:** 2023-07-05

**Authors:** Elli Mylona, Lisa Hefele, Nga Tran Vu Thieu, Tan Trinh Van, Chau Nguyen Ngoc Minh, Anh Tran Tuan, Abhilasha Karkey, Sabina Dongol, Buddha Basnyat, Phat Voong Vinh, Thanh Ho Ngoc Dan, Paula Russell, Richelle C Charles, Christopher M Parry, Stephen Baker

**Affiliations:** Cambridge Institute of Therapeutic Immunology and Infectious Disease, University of Cambridge School of Clinical Medicine, Cambridge, United Kingdom; Department of Medicine, University of Cambridge School of Clinical Medicine, Cambridge, United Kingdom; Department of Infection and Immunity, Luxembourg Institute of Health, Esch-sur-Alzette, Grand Duchy of Luxembourg; The Hospital for Tropical Diseases, Wellcome Trust Major Overseas Programme, Oxford University Clinical Research Unit, Ho Chi Minh City, Vietnam; The Hospital for Tropical Diseases, Wellcome Trust Major Overseas Programme, Oxford University Clinical Research Unit, Ho Chi Minh City, Vietnam; The Hospital for Tropical Diseases, Wellcome Trust Major Overseas Programme, Oxford University Clinical Research Unit, Ho Chi Minh City, Vietnam; The Hospital for Tropical Diseases, Wellcome Trust Major Overseas Programme, Oxford University Clinical Research Unit, Ho Chi Minh City, Vietnam; Oxford University Clinical Research Unit, Patan Academy of Health Sciences, Kathmandu, Nepal; Oxford University Clinical Research Unit, Patan Academy of Health Sciences, Kathmandu, Nepal; Oxford University Clinical Research Unit, Patan Academy of Health Sciences, Kathmandu, Nepal; The Hospital for Tropical Diseases, Wellcome Trust Major Overseas Programme, Oxford University Clinical Research Unit, Ho Chi Minh City, Vietnam; The Hospital for Tropical Diseases, Wellcome Trust Major Overseas Programme, Oxford University Clinical Research Unit, Ho Chi Minh City, Vietnam; Cambridge Institute of Therapeutic Immunology and Infectious Disease, University of Cambridge School of Clinical Medicine, Cambridge, United Kingdom; Department of Medicine, University of Cambridge School of Clinical Medicine, Cambridge, United Kingdom; Harvard Medical School, Massachusetts General Hospital, Boston, USA; Centre for Tropical Medicine, Oxford University, Oxford, United Kingdom; Clinical Sciences, Liverpool School of Tropical Medicine, Liverpool, United Kingdom; Cambridge Institute of Therapeutic Immunology and Infectious Disease, University of Cambridge School of Clinical Medicine, Cambridge, United Kingdom; Department of Medicine, University of Cambridge School of Clinical Medicine, Cambridge, United Kingdom; IAVI Human Immunology Laboratory, Imperial College London, London, UK

**Keywords:** enteric fever, IgG antibodies, longitudinal responses, serosurveillance

## Abstract

**Background:**

Enteric fever, caused by *Salmonella enterica* serovars Typhi and Paratyphi A, is a major public health problem in low- and middle-income countries. Moderate sensitivity and scalability of current methods likely underestimate enteric fever burden. Determining the serological responses to organism-specific antigens may improve incidence measures.

**Methods:**

Plasma samples were collected from blood culture-confirmed enteric fever patients, blood culture-negative febrile patients over the course of 3 months, and afebrile community controls. A panel of 17 *Salmonella* Typhi and Paratyphi A antigens was purified and used to determine antigen-specific antibody responses by indirect ELISAs.

**Results:**

The antigen-specific longitudinal antibody responses were comparable between enteric fever patients, patients with blood culture-negative febrile controls, and afebrile community controls for most antigens. However, we found that IgG responses against STY1479 (YncE), STY1886 (CdtB), STY1498 (HlyE), and the serovar-specific O2 and O9 antigens were greatly elevated over a 3-month follow up period in *S.* Typhi/*S.* Paratyphi A patients compared to controls, suggesting seroconversion.

**Conclusions:**

We identified a set of antigens as good candidates to demonstrate enteric fever exposure. These targets can be used in combination to develop more sensitive and scalable approaches to enteric fever surveillance and generate invaluable epidemiological data for informing vaccine policies.

**Clinical Trial Registration:**

ISRCTN63006567.

## Teaser

Poor enteric fever diagnostics limit our ability to estimate disease burden, which hinders vaccine introduction. We identified several protein and polysaccharide antigens that offer evidence of exposure and can aid serosurveillance studies.

Enteric fever, caused by the *Salmonella enterica* serovars Typhi and Paratyphi A, is a significant health burden in many low- and middle-income countries [1, 2]. The disease caused by *S*. Typhi and *S*. Paratyphi A is clinically indistinguishable [3] and has an estimated global annual incidence of >14 million cases with >135 000 deaths [1]. The ongoing introduction of typhoid conjugate vaccines (TCV), as well as improvement of sanitation in many endemic areas, has led to reduction in enteric fever incidence [1, 4, 5]. However, insufficient incidence data hinder accurate estimations of the disease burden in many countries, which prevents or delays vaccine implementation [6].

Enteric fever is diagnosed by isolating the infecting organism from blood, which permits antimicrobial susceptibility testing [4]. However, blood culture surveillance requires substantial laboratory infrastructure, has only moderate sensitivity, is reliant on health care-seeking behavior to generate adequate incidence data, and can detect only those with disease. Consequently, blood culture dramatically underestimates the actual burden of symptomatic and asymptomatic enteric fever [7–9]. A more passive approach to febrile disease surveillance, such as population-based serosurveillance, can more accurately estimate the force of infection by measuring serological responses to antigens that are specific to the selected organism [8, 10]. However, serosurveillance for enteric fever historically has suffered from a lack of reliable pathogen-specific antigens and is conventionally performed by measuring the serological response against the Vi polysaccharide, lipopolysaccharide O-antigen, and/or flagellar H-antigens [10–12]. Such an approach is compromised by poor seroconversion against Vi, which is also the principal component of TCV. Furthermore, endemic communities often have high preexisting antibodies against O- and H-antigens, which are associated with other *Salmonella* [10–14].

Here, by assessing the longitudinal antibody responses to multiple antigens in a cohort of Nepali patients presenting with febrile disease we aimed to generate new methods for enteric fever surveillance. We tested several conventional antigens in addition to various protein antigens previously identified as being potentially informative for identification of people having an immunological interaction with *S*. Typhi [14–16]. We measured immunoglobulin G (IgG) antibody responses over a 3-month follow-up period in patients with culture-confirmed *S*. Typhi and *S.* Paratyphi A infections and febrile patients with a negative blood culture.

## METHODS

### Ethics

The original treatment trial from which the plasma samples originated was granted ethical approval by the Nepal Health Research Council, Kathmandu, Nepal and the Oxford Tropical Research Ethics Committee, Oxford, UK [17].

### Study Design and Population

All plasma samples originated from an open-label, randomized, controlled trial, comparing gatifloxacin and ofloxacin for the treatment of uncomplicated enteric fever conducted at Patan Hospital, Nepal (trial registered as ISRCTN63006567) [17]. Patients were randomly assigned to a 7-day course of treatment [17]. Blood was collected in EDTA tubes on day 1 (day of enrolment), day 8, day 28 (month 1), and day 90 (month 3), and was immediately separated into cells and plasma. Only plasma samples from patients that presented with 72–96 hours of fever (day 1) were analyzed. Longitudinal samples were collected from 103 and 61 culture-confirmed *S*. Typhi and *S*. Paratyphi A patients, respectively. Comparative samples were collected from 322 culture-negative febrile patients (FCN) (Table 1 and Supplementary Table 1) and a single time-point plasma sample was collected from 49 afebrile, community controls (CCs).

**Table 1.** Participant Characteristics

Characteristic	Community Controls n = 49	All Patient Groups n = 486	Patient Groups		
			FCN	SPA	ST
			n = 322	n = 61	n = 103
Sex, No. (%)					
Female	NA	166 (34.2)	111 (34.5)	23 (37.7)	32 (31.1)
Male	NA	320 (65.9)	211 (65.5)	38 (62.3)	71 (68.9)
Age, mean (SD)	NA	17.3 (9.8)	17.9 (10.6)	16.8 (8.1)	15.6 (7.9)
NA	…	3	1	0	2

Abbreviations: FCN, culture-negative febrile patients; NA, not available; SD, standard deviation; SPA, *Salmonella* Paratyphi A-confirmed patients; ST, *Salmonella* Typhi-confirmed patients.

### Protein Expression

Protein expression was induced in *Escherichia coli* BL21(DE3) pLysS harboring each of the plasmid constructs (pEK90–pEK109; Supplementary Table 2) with 0.1 mM isopropyl-β-D-thiogalactoside (IPTG) (Sigma Aldrich) for 3 hours at 24°C, before harvesting bacteria by centrifugation (5000*g*, 4°C). For soluble proteins, pellets were resuspended by sonication in 50 mM phosphate buffer (pH 8) containing 300 mM NaCl/10 mM imidazole, and pelleted by centrifugation (16 000*g*, 4°C, 30 minutes). Proteins were bound on nickel-coated agarose beads (Ni-NTA, Invitrogen) by rocking the supernatants at 4°C for 2 hours. The beads were loaded onto gravity flow columns (Qiagen), washed with 20 mM imidazole in phosphate buffer, and proteins were eluted with 250 mM imidazole in phosphate buffer. For insoluble proteins, bacterial pellets were first incubated in 8 M urea (pH 7.8) in 20 mM sodium phosphate/500 mM NaCl. Proteins were eluted with 4 M urea (pH 3) in 20 mM sodium phosphate buffer/500 mM NaCl and recovered in 50 mM sodium phosphate/500 mM NaCl.

### ELISA to detect IgG in Human Plasma against Salmonella antigens

Enzyme-linked immunosorbent assays (ELISAs) to detect IgG in human plasma samples against selected antigens (Supplementary Table 3) were performed as previously described [16]. Briefly, antigens were prepared in 50 mM carbonate bicarbonate buffer at 1 μg/mL for proteins and Vi, and at 15 μg/mL for O2 and O9. Flat-bottom, 96-well ELISA plates (Nunc 2404, Thermo Scientific) were coated with 100 μL per well of each antigen overnight. Coated plates were washed and blocked with 5% fat-free milk in phosphate-buffered saline for 1 hour before being washed and incubated with 100 μL per well of a 1:200 dilution of plasma at ambient temperature for 2 hours. Plates were washed and incubated with 100 μL per well of alkaline phosphatase-conjugated anti-human IgG at ambient temperature for 1 hour, before treating with phosphate-buffered saline *p*-nitrophenyl phosphate (SigmaFAST N1891; Sigma-Aldrich) substrate for 30 minutes at ambient temperature. The absorbance was read at 405 and 490 nm using a microplate reader (Biorad). The end point absorbance was read as positive when its value was greater than that of the blank wells plus 2 times the standard deviation. ELISA units (EU) were expressed relative to standard curves, with best 4-parameter fit determined by a modified Hill plot; standard curves were constructed for IgM and IgG independently. The reference standard of each ELISA was a pool of known antigen-specific antibody human plasma collected from a bank of Nepalese plasma. The standard curves were then generated from corresponding optical densities (ODs) of a (10-point) 2-fold serial dilution of the plasma pool. The definition of 1 EU was the reciprocal of the dilution of the standard plasma that gave an OD value equal to 1 within the assay.

### Data Analysis

Data analyses were conducted in R software [18] (for details please refer to the Supplement Material). The antibody responses measured by ELISA were log transformed. The antibody responses for each time point, antigen, and patient group were fitted to a LOESS smooth function. After analysis of normality of independent variables in the different groups (Shapiro-Wilk test) and homogeneity of the variances between the groups (Levene test), nonparametric statistical tests were employed.

Differences between time points within the patient groups were assessed by the Friedman test followed by Wilcoxon test for paired data with Bonferroni correction. A balanced design is necessary to conduct Friedman test, therefore we had to exclude participants without samples at each time point. Kruskal-Wallis test followed by Wilcoxon test to calculate pairwise comparisons between group levels with Bonferroni correction were used to test the significance of the differences between the antibody titer measured at different time points within each group. The Spearman correlation coefficients were calculated to measure the correlation between the antibody responses to the different antigens at the different sampling time points. A *P* value <.05 was considered statistically significant.

## RESULTS

### Longitudinal Responses

**Figure 1.**
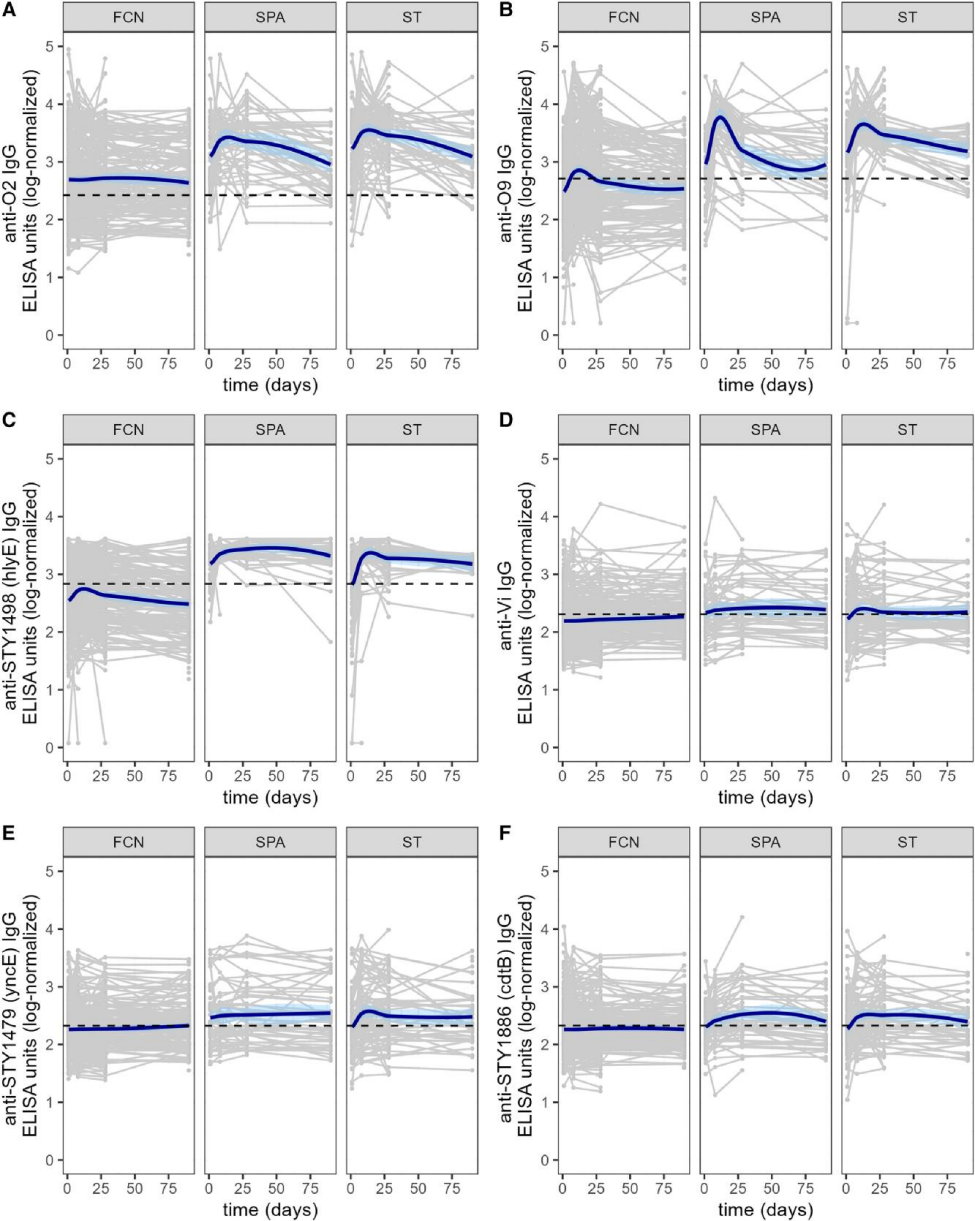
The longitudinal IgG responses against *Salmonella* Typhi or *S*. Paratyphi A purified antigens in a Nepali cohort of febrile patients. IgG antibody responses against O2 (*A*), O9 (*B*), STY1498 (*C*), Vi (*D*), STY1479 (*E*), and STY1886 (*F*) over the course of 3 months in different patient groups. Grey lines show antibody trajectories in individual patients and the solid bold line shows the fitted LOESS smooth function. Dashed lines represent the mean titer value within the control group of afebrile, community control samples for the respective antibody. Abbreviations: ELISA, enzyme-linked immunosorbent assay; FCN, culture negative febrile patients; IgG, immunoglobulin G; SPA, *S*. Paratyphi A confirmed patients; ST, *S*. Typhi-confirmed patients.

We selected a panel of 17 antigens including Vi, O2, O9, and other protein antigens we hypothesized to show a differential response during the natural immunological history of enteric fever [14–16] (Supplementary Table 3). IgG antibodies against these antigens were determined in plasma from *S*. Typhi, *S*. Paratyphi A, and FCN patients over the course of 3 months. We additionally measured the same responses in 49 afebrile CCs as baseline measurements; these data were not considered in the longitudinal analysis. The antibody responses against each of the antigens were highly variable between individuals at all time points (Figure 1 and Supplementary Figures 1 and 2). The longitudinal antibody trajectories for each patient group were fitted to LOESS smooth functions to determine changes over time. Overall, the majority of antigen-specific IgG profiles generated comparable curves between the enteric fever (*S*. Typhi and *S*. Paratyphi A) and FCN patients and were unchanged from baseline (Supplementary Figures 1 and 2), signifying that the longitudinal IgG responses against these antigens were largely uninformative.

We identified a subset of 5 antigens that exhibited distinct antibody dynamics between the *S*. Typhi/*S*. Paratyphi A and the FCN patients (Figure 1). The longitudinal IgG responses against O2, O9, and STY1498 (HlyE) generated the most distinct fitted curves and were markedly elevated in the *S*. Typhi and *S*. Paratyphi A groups in comparison to the FCN patients (Figure 1*A*–1*C*). Additionally, the IgG responses against STY1479 (YncE) and STY1886 (CdtB) in the *S*. Paratyphi A and *S*. Typhi groups were higher than in the FCN group and remained above baseline over the 3-month sampling period (Figure 1*E* and 1*F*). Notably, anti-STY1767 IgG antibodies were consistently higher in *S*. Paratyphi A patients than those in the FCN group; this was not replicated in the *S*. Typhi group (Supplementary Figure 2*E*). Lastly, the antibody dynamics against Vi were not substantially different in the *S*. Paratyphi A group compared to the FCN group, while the Vi IgG trajectory elaborated an early peak in the *S*. Typhi group but returned rapidly to baseline (Figure 1*D*).

### Peak Antibody Responses

**Figure 2.**
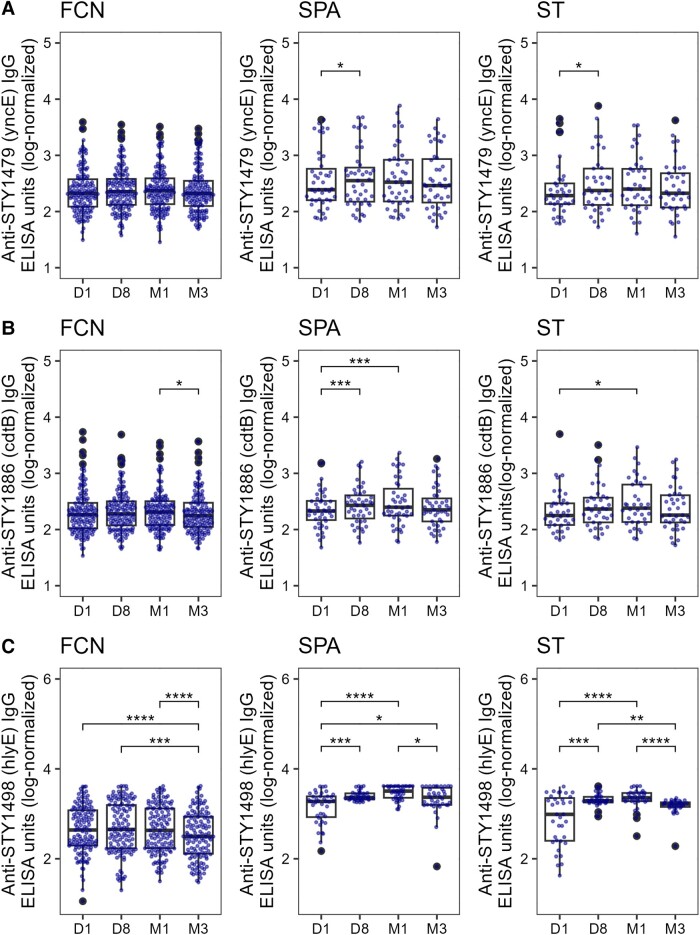
The distribution of serum IgG titers in a Nepali cohort of enteric fever patients and febrile culture-negative controls. Boxplots showing IgG titers in FCN, SPA, or ST against STY1479 (*A*), STY1886 (*B*), and STY1498 (*C*) antigens over the course of 3 months. Each dot shows the antibody titer of an individual sample on day 1 (D1), day 8 (D8), month 1 (M1), and month 3 (M3). Differences between time points were assessed using Friedman test followed by pairwise comparisons using Wilcoxon signed-rank tests. *P* values were adjusted using the Bonferroni multiple testing correction method. *P < .05, **P < .01, ***P < .001, ****P < .0001. Abbreviations: ELISA, enzyme-linked immunosorbent assay; FCN, culture negative febrile patients; IgG, immunoglobulin G; SPA, *Salmonella* Paratyphi A confirmed patients; ST, *S*. Typhi-confirmed patients. The box extends from 1st to 3rd quartile; IQR. The minimum/maximum whisker values are calculated as Q1/Q3 -/+ 1.5 * IQR.

**Figure 3.**
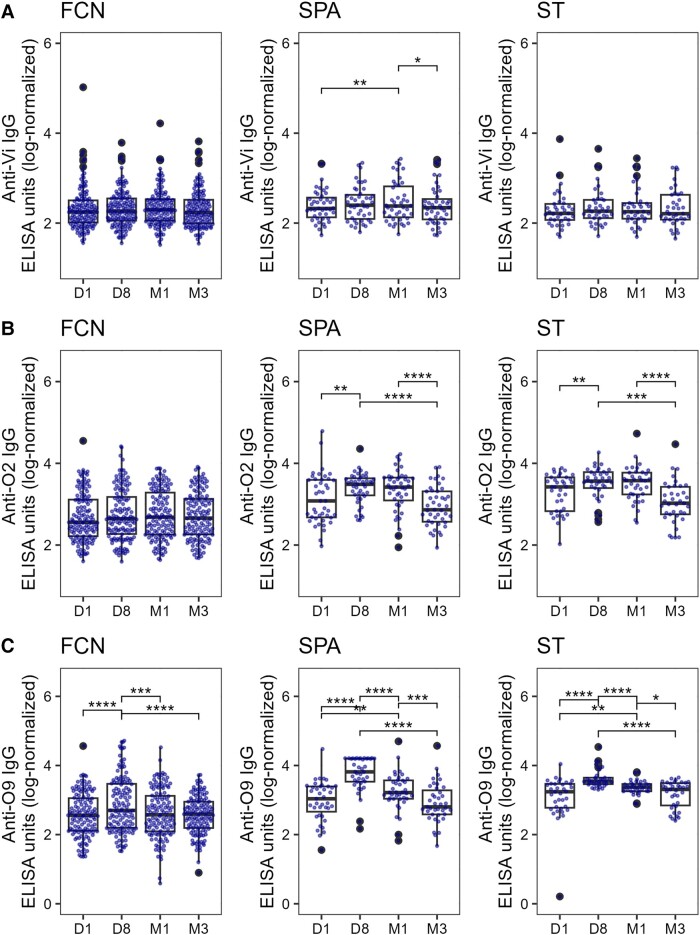
The distribution of serum IgG titers in a Nepali cohort of enteric fever patients and febrile culture-negative controls. Boxplots showing IgG titers in plasma from FCN, SPA, or ST against Vi (*A*), O2 (*B*), and O9 (*C*) antigens over the course of 3 months. Each dot shows the antibody titer of an individual sample on day 1 (D1), day 8 (D8), month 1 (M1), and month 3 (M3). Differences between time points were assessed using Friedman test followed by pairwise comparisons using Wilcoxon signed-rank tests. *P* values were adjusted using the Bonferroni multiple testing correction method. *P < .05, **P < .01, ***P < .001, ****P < .0001. Abbreviations: ELISA, enzyme-linked immunosorbent assay; FCN, culture negative febrile patients; IgG, immunoglobulin G; SPA, *Salmonella* Paratyphi A confirmed patients; ST, *S*. Typhi-confirmed patients. The box extends from 1st to 3rd quartile; IQR. The minimum/maximum whisker values are calculated as Q1/Q3 -/+ 1.5 * IQR.

We next disaggregated the antibody trajectories into their single time points. The antibody responses against the majority of antigens remained unchanged or slightly reduced in the FCN group over time (Figures 2, Figure 3, and Supplementary Figures 3–5), with the notable exception of O9, which peaked on day 8 and had declined by month 1 (Figure 3*C*).

The antibody values against STY1886 (CdtB), SY1498 (HlyE), O2, and O9 in the *S*. Typhi and/or *S*. Paratyphi A patient groups increased significantly (*P* < .05) from day 1 to day 8 and remained elevated at month 1 (Figure 2 and Figure 3). Similarly, a significant (*P* < .05) but less prominent increase in antibody response was also observed at day 8 and month 1 in comparison to day 1 against all other antigens tested in the *S*. Paratyphi A patient group (Supplementary Figures 3–5), with the notable exception of Vi (Figure 3*A*). The IgG responses against STY1612 (Supplementary Figure 3*B*), STY4539 (Supplementary Figure 3*C*), STY1086 (Supplementary Figure 4*B*), and STY1703 (Supplementary Figure 5*A*) peaked at day 8 in the *S*. Typhi group, with anti-STY1086 and anti-STY1703 IgG remaining high up to month 1. Additionally, the anti-Vi IgG responses remained stable with time in the *S*. Typhi group and were comparable to those measured at day 1 (Figure 3*A*). The majority of IgG trajectories exhibited a notable decline by month 3, with anti-STY1479 (YncE; Figure 2*A*), anti-STY1498 (HlyE; Figure 2*C*), anti-O2 (Figure 3*B*), and anti-O9 (Figure 3*C*) having a comparable decrease in both the *S*. Typhi and *S*. Paratyphi A groups.

### Correlation Between Antibody Responses

**Figure 4.**
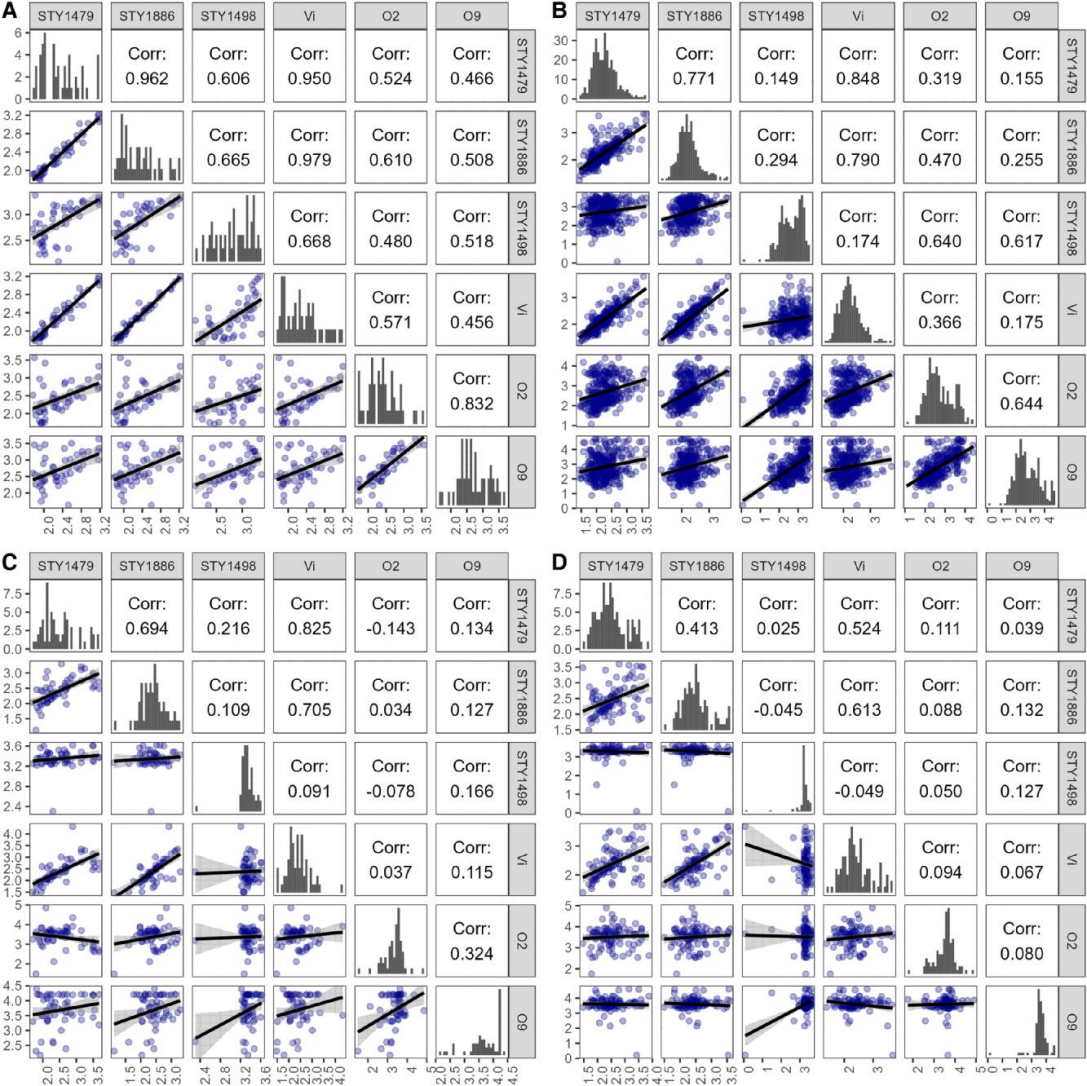
Correlation between antibody responses to selected *Salmonella* antigens on day 8. Spearman correlation among antigen-specific antibodies in community controls (*A*), from febrile, culture-negative patients (*B*), *Salmonella* Paratyphi A confirmed patients (*C*), and *S*. Typhi confirmed patients (*D*). Histograms show the distribution of the of each antigen-specific IgG antibody measurement on the diagonal. Scatterplots below the diagonal represent the correlation of IgG measurements of the 2 antigens on a right angle to the plots. The numerals above the diagonal depict the Spearman correlation coefficient (*ρ*) values of the mirrored plots.

Spearman correlation coefficients were calculated to determine the nature of the correlations between antibody responses on day 8 (the peak of most antibody trajectories) within each patient group (Figure 4 and Supplementary Figures 6–9). The antibody responses to all protein antigens and Vi demonstrated a moderate to strong correlation with each other (*ρ* > 0.6) within the CC group (Figure 4*A* and Supplementary Figure 6). The anti-O2 and anti-O9 responses correlated less well (*ρ* = 0.4–0.7) to the majority of responses to other antigens (Figure 4*A* and Supplementary Figure 6). In comparison, the antibody responses in the FCN group correlated moderately with each other (*ρ* > 0.5), with the notable exceptions of anti-STY1498 (HlyE), anti-O2, and anti-O9, which correlated with each other but exhibited a weak correlation to all other antigen-specific responses (*ρ* < 0.5) (Figure 4*B* and Supplementary Figure 7).

The day 8 antibody responses against all protein antigens and the Vi correlated moderately to strongly to each other in the *S*. Paratyphi A (Figure 4*C* and Supplementary 8) and *S*. Typhi (*ρ* > 0.4) (Figure 4*D* and Supplementary Figure 9) groups, with the notable exception of STY1498 (HlyE; Figure 4*C*–4*D*, and Supplementary Figures 8 and 9), which correlated poorly to all antibody responses (*ρ* < 0.2) due to its unique trajectory (Figure 1). Among *S*. Paratyphi A patients, *ρ* values of 0.6 and 0.7 between anti-Vi and anti-STY1479 (YncE) and anti-STY1886 (CdtB) antibodies, respectively, were observed (Figure 4*C*). The anti-O2 and anti-O9 IgG responses correlated poorly to all other antigens and to each other in the *S*. Paratyphi A and *S*. Typhi groups, with *ρ* values of 0.32 and 0.08, respectively (Figure 4*C*–4*D* and Supplementary Figures 8 and 9).

### Longitudinal Comparison of Antibody Titers

**Figure 5.**
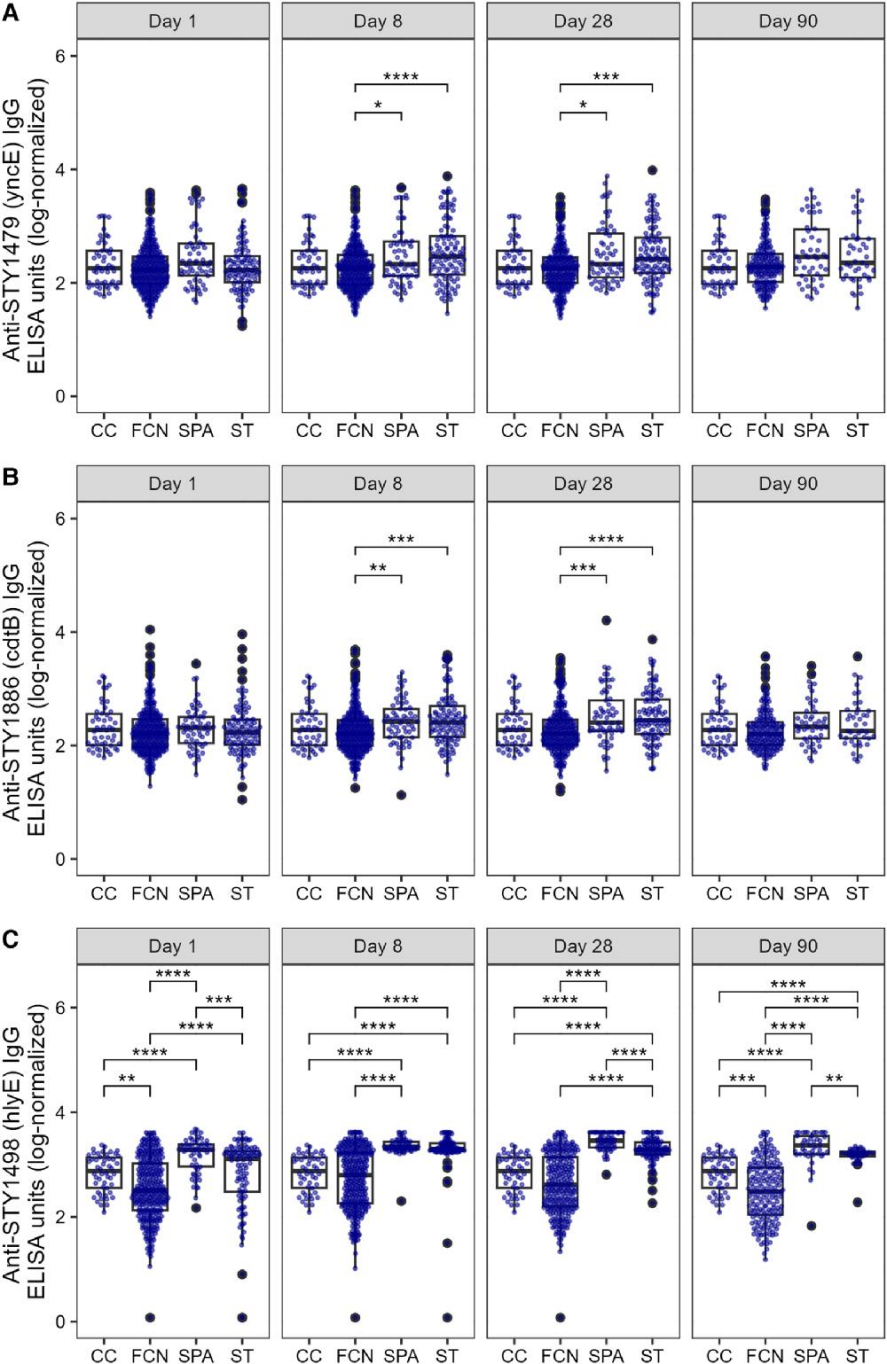
The distribution of serum IgG titers in a Nepali cohort of febrile patients and controls. Boxplots showing IgG titers in plasma from CC, FCN infected with an unidentified pathogen, SPA, or ST against STY1479 (*A*), STY1886 (*B*), and STY1498 (*C*) antigens over the course of 3 months. Differences between time points were assessed first using Kruskal-Wallis test followed by pairwise comparisons using Wilcoxon signed-rank tests. *P* values were adjusted using the Bonferroni multiple testing correction method. *P < .05, **P < .01, ***P < .001, ****P < .0001. Abbreviations: CC, community control; ELISA, enzyme-linked immunosorbent assay; FCN, culture negative febrile patients; IgG, immunoglobulin G; SPA, *Salmonella* Paratyphi A confirmed patients; ST, *S*. Typhi-confirmed patients. The box extends from 1st to 3rd quartile; IQR. The minimum/maximum whisker values are calculated as Q1/Q3 -/+ 1.5 * IQR.

**Figure 6.**
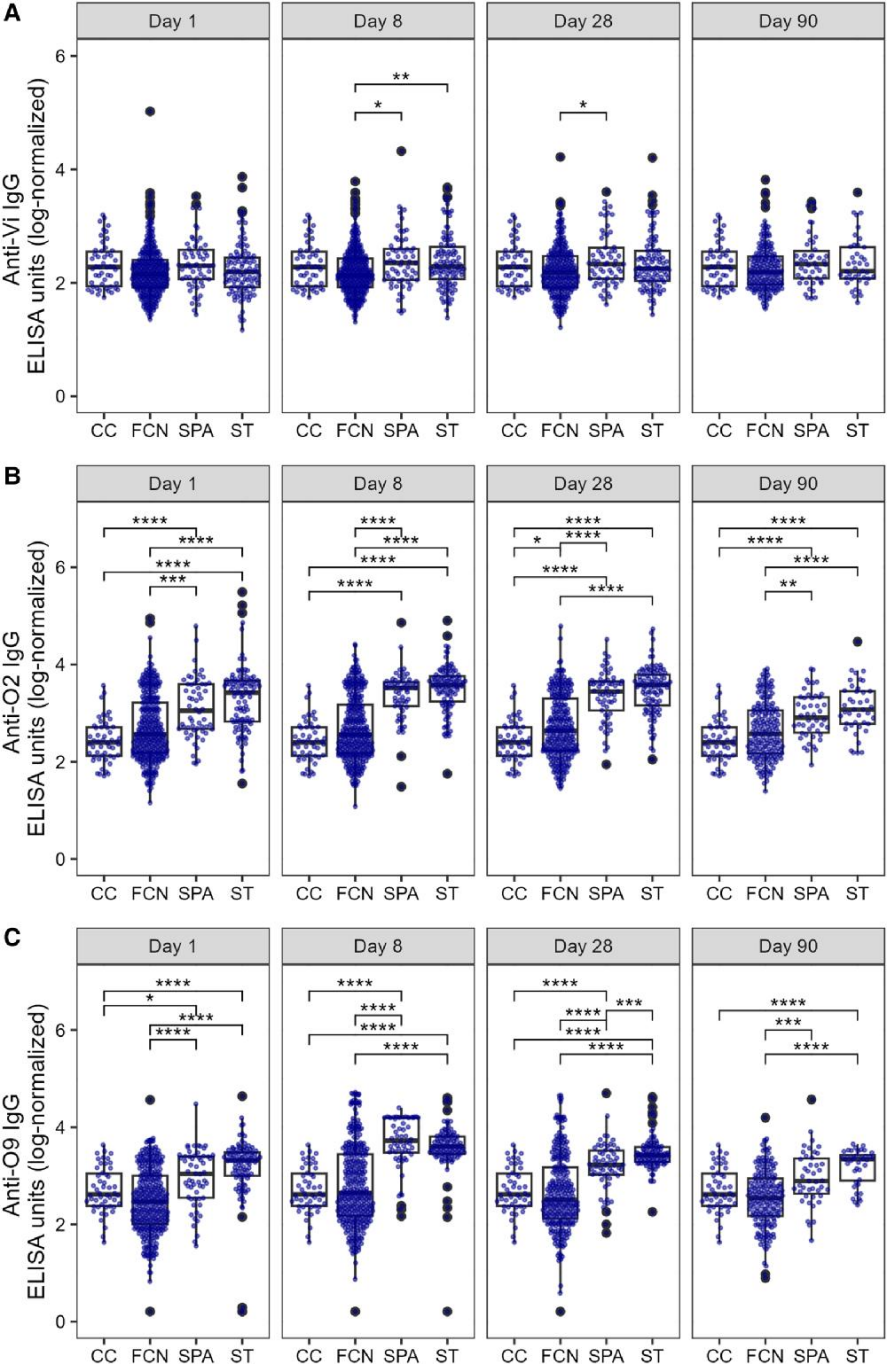
The distribution of serum IgG titers in a Nepali cohort of febrile patients and controls. Boxplots showing IgG titers in plasma from CC, FCN infected with an unidentified pathogen, SPA, or ST against Vi (*A*), O2 (*B*), and O9 (*C*) antigens over the course of 3 months. Differences between time points were assessed first using Kruskal-Wallis test followed by pairwise comparisons using Wilcoxon signed-rank tests. *P* values were adjusted using the Bonferroni multiple testing correction method. *P < .05, **P < .01, ***P < .001, ****P < .000. Abbreviations: ELISA, enzyme-linked immunosorbent assay; FCN, culture negative febrile patients; IgG, immunoglobulin G; SPA, *Salmonella* Paratyphi A confirmed patients; ST, *S*. Typhi-confirmed patients. The box extends from 1st to 3rd quartile; IQR. The minimum/maximum whisker values are calculated as Q1/Q3 -/+ 1.5 * IQR.

We lastly compared the median antibody responses between each patient group at each time point and to single time point CC and FCN plasma (Figure 5, Figure 6, and Supplementary Figures 10–12). All antibody responses, other than anti-STY1498 (HlyE; Figure 5*C*), anti-O2 (Figure 6*B*), and anti-O9 (Figure 6*C*), were comparable between groups on day 1. The antibody concentrations against STY1498 (HlyE; Figure 5*C*) and STY1703 (Supplementary Figure 12*A*) were significantly higher on day 8, month 1, and month 3 in the *S*. Typhi and/or *S*. Paratyphi A groups compared to both the CC and the FCN controls (*P* < .05 for all comparisons). The median IgG responses against STY1479 (YncE) and STY1886 (CdtB) in *S*. Typhi and *S*. Paratyphi A groups were higher in comparison to the FCN group on day 8 and month 1 (Figure 5*A* and 5*B*). Similarly, anti-O2 (Figure 6*B*) and anti-O9 (Figure 6*C*) IgG responses were elevated on day 8, month 1, and month 3 in the *S*. Typhi and *S*. Paratyphi A groups. Notably, IgG against the serovar-specific O-antigen, along with anti-STY1498 (HlyE) IgG, were higher in the *S*. Typhi and *S*. Paratyphi A groups than the CC and FCN groups on day 1 (Figure 5*C* and Figure 6*A* and 6*B*). The median IgG values against the Vi were slightly but significantly increased on day 8 in both *S*. Paratyphi A and *S*. Typhi groups compared to the FCN group, but these were comparable in convalescent samples at month 3 (Figure 6*A*).

## DISCUSSION

Serosurveillance for enteric fever is impaired by a lack of specific and sensitive informative antigens and methods. Here, we determined the antibody responses in enteric fever patients (blood-confirmed *S*. Typhi or *S*. Paratyphi A-infected) against 17 antigens and compared them to the responses in febrile patients with a negative blood culture and community controls (baseline). We tested protein antigens that we had previously considered as candidates for acute diagnostic markers and are largely conserved between *S.* Typhi and *S.* Paratyphi A, as well as the Vi, O2, and O9 antigens, that are commonly used to identify enteric fever patients, to identify biomarkers for enteric fever exposure.

The longitudinal antibody responses to most antigens tested were not significantly different between *S*. Typhi/*S*. Paratyphi A patients, FCN controls, or community controls. We identified a subset of protein antigens (STY1479 or YncE, STY1886 or CdtB, and STY1498 or HlyE) that generated distinct antibody trajectories in both *S*. Typhi and *S*. Paratyphi A groups compared to FCN patients, indicative of potential seroconversion. While antibodies against other antigens, such as STY1086, elaborated a similar increase with time, this change was small and likely of limited utility in serosurveillance. Anti-STY1767 antibodies were consistently higher in *S*. Paratyphi A compared to FCN patients, while *S*. Typhi patients showed such an increase only later. While anti-STY1767 antibodies in *S*. Typhi and *S*. Paratyphi A groups did not differ significantly, further investigations will need to determine whether responses to STY1767 can contribute to distinguishing the 2 infections. We additionally compared the antibody responses to Vi, O2, and O9 in enteric fever patients to febrile and afebrile controls. The early peak response observed against O9, and O2 (as well as HlyE) are likely a recall response from prior exposure to *S*. Typhi and/or *S*. Paratyphi A for these immunodominant antigens. Cross-reactivity with other Enterobacteriaceae should be considered and evaluated to ensure specificity of these antigens. In this analysis, we did see cross-reactivity between sera from *S*. Typhi and *S*. Paratyphi patients to O2 and O9 likely due to shared O12 antigen, which is present in both O-antigen conformations. The O12 is also present in a number of invasive nontyphoidal *Salmonella* (NTS) and, therefore, these antigens may be less useful if used alone as a specific marker for enteric fever in populations where the burden of invasive NTS is high, such as Africa. Alternatively, the fact that *S*. Typhi patients generate a measurable antibody response against O2 from *S*. Paratyphi A (and vice versa for O9) may implicate anti-O12 responses, which is conserved between the 2 O-antigen conformations. Additionally, the baseline antibody responses against Vi in enteric fever patients suggest that anti-Vi antibodies are circulating in this community and the Vi is not suitable as a marker of enteric fever exposure. The use of Vi in this context is likely to be further hampered by ongoing TCV vaccination schemes and it is known that responses to Vi cannot distinguish between natural infection and vaccine-mediated exposure [19].

The protein antigens exhibiting the greatest potential for measuring enteric fever exposure play differing roles during infection. STY1886 (CdtB) is a component of typhoid toxin, which has a predicted role in symptom manifestation, eliminating host immune cells, and damaging host DNA [20–23]. The typhoid toxin is rarely found in NTS [24, 25]. While its precise role in virulence and clinical presentation remains undefined [21], antibody responses against CdtB in enteric fever patients have been described previously [14, 16, 26]. STY1498 (HlyE) is a pore-forming toxin, which is absent from most NTS but present in other Enterobacteriaceae including *E. coli* and *Shigella* [27–29]. Like CdtB, the precise role of HlyE in virulence remains unclear; studies have suggested that HlyE promotes chronic infection, host cell invasion, and organ colonization [30]. Antibodies against HlyE have been detected in plasma and mucosal responses in acute typhoid patients and thus HlyE has been identified elsewhere as a potential target for enteric fever serodiagnosis and serosurveillance [10, 14, 26, 31]. Lastly, STY1479 (YncE) is a putative ATP-binding protein with unknown function but induced upon iron restriction in *E. coli* and involved in iron acquisition. In enteric fever, YncE has been identified as a potential marker for chronic carriers given its high immunoreactivity in carriers compared to acute or convalescent patients [15]. In our study, anti-YncE antibody responses were higher in enteric fever patients compared to febrile controls even in the acute stages of infection.

This study has limitations. Even though we could determine potential seroconversion in a subset of antigen-specific responses, cross-reactivity with other Enterobacteriaceae may remain a problem. Additionally, while beyond the scope of this study, these antigens still cannot as yet distinguish between *S*. Typhi and *S*. Paratyphi A infection and development of models to predict the cause of infection is required. Given the limited sensitivity of blood culture, it is likely that some of the FCN patients were indeed enteric fever patients, and thus elevated antibody responses in this group were observed. Future studies will also need to record responses by other antibody subtypes that are relevant, as reported previously [32], as well as to include chronic carriers to confirm the use of these antigens for the purpose of identifying those individuals.

In summary, by investigating the serologic dynamics of enteric fever via determining the longitudinal responses to an array of antigens in a relatively large endemic population and comparing them to endemic febrile and healthy control groups, we identified protein antigens (ie, HlyE, CdtB, and YncE) that are akin to findings from other groups, supporting their role as discriminatory markers for detecting enteric fever patients [10, 31–34], in line with them being largely specific to typhoidal *Salmonella* serovars. Further studies are underway to develop methods to distinguish between *S*. Typhi and *S*. Paratyphi A patients [10], as well as to optimally multiplex antigens to increase accuracy of serosurveillance and diagnostics even at early stages of infection. Whilst blood culture is a highly useful diagnostic method during symptomatic stages of enteric fever, serosurveillance is straightforward, scalable, and informative for vaccine policy. The data presented here can be used for developing mathematical inferences that can be applied to single samples (including dried blood spots assays), thereby creating a cost-effective and sensitive surveillance tool that can be made mobile and be used in endemic locations with minimal infrastructure. We conclude that the antigens and assay described here may offer a substantial change in approaches to enteric fever surveillance, contributing to the need for more of efficient and cost-effective data collection regarding enteric fever epidemiology.

## Supplementary Data

Supplementary materials are available at *The Journal of Infectious Diseases* online. Consisting of data provided by the authors to benefit the reader, the posted materials are not copyedited and are the sole responsibility of the authors, so questions or comments should be addressed to the corresponding author.

## Supplementary Material

jiad242_Supplementary_Data

## Notes

***Author contributions.*** E. M. and L. H. wrote the manuscript and performed analysis. N. T. V. T. wrote the manuscript, ran experiments, and performed analysis. T. T. V., C. N. N. M., P. V. V., and T. H. N. D. ran experiments. A. T. T. performed analysis. A. K., S. D., and B. B. provided data. P. R. generated data. R. C. C. provided reagents. C. M. P. performed supervision. S. B. contributed the concept, performed supervision, and wrote the manuscript.

***Acknowledgments.*** The authors acknowledge the staff contributing to the recruitment and sampling of enteric fever patients at Patan Hospital, Kathmandu, and the patients themselves.

***Disclaimer***. The funders had no role in the design and conduct of the study; collection, management, analysis, and interpretation of the data; preparation, review, or approval of the manuscript; and decision to submit the manuscript for publication.

***Financial support.*** This work was supported by the Wellcome Trust (grant number 215515/Z/19/Z senior research fellowship to S. B.); and the National Institutes of Allergy and Infectious Diseases (grant number R01 AI134814 to R. C. C.).

## References

[1] Stanaway JD, Reiner RC, Blacker BF, et al The global burden of typhoid and paratyphoid fevers: a systematic analysis for the global burden of disease study 2017. Lancet Infect Dis 2019; 19:369–81. 30792131 10.1016/S1473-3099(18)30685-6 PMC6437314

[2] Tamrakar D, Vaidya K, Yu AT, et al Spatial heterogeneity of enteric fever in 2 diverse communities in Nepal. Clin Infect Dis 2020; 71:S205–13. 33258932 10.1093/cid/ciaa1319 PMC7705881

[3] Maskey AP, Day JN, Tuan PQ, et al *Salmonella enterica* serovar Paratyphi A and *S. enterica* serovar Typhi cause indistinguishable clinical syndromes in Kathmandu, Nepal. Clin Infect Dis 2006; 42:1247–53. 16586383 10.1086/503033

[4] Crump JA, Sjölund-Karlsson M, Gordon MA, Parry CM. Epidemiology, clinical presentation, laboratory diagnosis, antimicrobial resistance, and antimicrobial management of invasive *Salmonella* infections. Clin Microbiol Rev 2015; 28:901–37. 26180063 10.1128/CMR.00002-15 PMC4503790

[5] Birkhold M, Mwisongo A, Pollard AJ, Neuzil KM. Typhoid conjugate vaccines: advancing the research and public health agendas. J Infect Dis 2021; 224:S781–7. 34528085 10.1093/infdis/jiab449 PMC8687070

[6] Record WHO-WE, 2008 undefined. Typhoid vaccines: WHO position paper. apps.who.int 2008. https://www.who.int/teams/health-product-policy-and-standards/standards-and-specifications/vaccinestandardization/typhoid-fever

[7] Parry CM, Wijedoru L, Arjyal A, Baker S. The utility of diagnostic tests for enteric fever in endemic locations. Expert Rev Anti Infect Ther 2011; 9:711–25. 21692675 10.1586/eri.11.47

[8] Carey ME, MacWright WR, Im J, et al The Surveillance for Enteric Fever in Asia Project (SEAP), Severe Typhoid Fever Surveillance in Africa (SETA), Surveillance of Enteric Fever in India (SEFI), and Strategic Typhoid Alliance Across Africa and Asia (STRATAA) population-based enteric fever studies: a review of methodological similarities and differences. Clin Infect Dis 2020; 71:S102–10. 32725221 10.1093/cid/ciaa367 PMC7388711

[9] Antillon M, Saad NJ, Baker S, Pollard AJ, Pitzer VE. The relationship between blood sample volume and diagnostic sensitivity of blood culture for typhoid and paratyphoid fever: a systematic review and meta-analysis. J Infect Dis 2018; 218:S255–67. 30307563 10.1093/infdis/jiy471 PMC6226661

[10] Aiemjoy K, Seidman JC, Saha S, et al Estimating typhoid incidence from community-based serosurveys: a multicohort study. Lancet Microbe 2022; 3:e578–87. 35750069 10.1016/S2666-5247(22)00114-8 PMC9329131

[11] House D, Wain J, Ho VA, et al Serology of typhoid fever in an area of endemicity and its relevance to diagnosis. J Clin Microbiol 2001; 39:1002–7. 11230418 10.1128/JCM.39.3.1002-1007.2001 PMC87864

[12] House D, Ho VA, Diep TS, et al Antibodies to the Vi capsule of *Salmonella* Typhi in the serum of typhoid patients and healthy control subjects from a typhoid endemic region. J Infect Dev Ctries 2008; 2:308–12. 19741294 10.3855/jidc.227

[13] House D, Chinh NT, Diep TS, et al Use of paired serum samples for serodiagnosis of typhoid fever. J Clin Microbiol 2005; 43:4889–90. 16145168 10.1128/JCM.43.9.4889-4890.2005 PMC1234064

[14] Liang L, Juarez S, Nga TVT, et al Immune profiling with a *Salmonella* Typhi antigen microarray identifies new diagnostic biomarkers of human typhoid. Sci Rep 2013; 3:1043. 23304434 10.1038/srep01043 PMC3540400

[15] Charles RC, Sultana T, Alam MM, et al Identification of immunogenic *Salmonella enterica* serotype Typhi antigens expressed in chronic biliary carriers of *S.* Typhi in Kathmandu, Nepal. PLoS Negl Trop Dis 2013; 7:e2335. 23936575 10.1371/journal.pntd.0002335 PMC3731212

[16] Tran Vu Thieu N, Trinh Van T, Tran Tuan A, et al An evaluation of purified *Salmonella* Typhi protein antigens for the serological diagnosis of acute typhoid fever. J Infect 2017; 75:104–14. 28551371 10.1016/j.jinf.2017.05.007 PMC5522525

[17] Koirala S, Basnyat B, Arjyal A, et al Gatifloxacin versus ofloxacin for the treatment of uncomplicated enteric fever in Nepal: an open-label, randomized, controlled trial. PLoS Negl Trop Dis 2013; 7:e2523. 24282626 10.1371/journal.pntd.0002523 PMC3837022

[18] R Core Team . R: A Language and Environment for Statistical Computing. Vienna, Austria: R Foundation for Statistical Computing, 2014.

[19] Watson CH, Baker S, Lau CL, et al A cross-sectional seroepidemiological survey of typhoid fever in Fiji. PLoS Negl Trop Dis 2017; 11:e0005786. doi: 10.1371/journal.pntd.0005786. PMID: 28727726; PMCID: PMC5549756. 28727726 PMC5549756

[20] Song J, Gao X, Galán JE. Structure and function of the *Salmonella* Typhi chimaeric A2B5 typhoid toxin. Nature 2013; 499:350–4. 23842500 10.1038/nature12377 PMC4144355

[21] Gibani MM, Jones E, Barton A, et al Investigation of the role of typhoid toxin in acute typhoid fever in a human challenge model. Nat Med 2019; 25:1082–8. 31270506 10.1038/s41591-019-0505-4 PMC6892374

[22] Ibler AEM, ElGhazaly M, Naylor KL, Bulgakova NA, El-Khamisy S F, Humphreys D. Typhoid toxin exhausts the RPA response to DNA replication stress driving senescence and *Salmonella* infection. Nat Commun 2019; 10:4040. 31492859 10.1038/s41467-019-12064-1 PMC6731267

[23] Spanò S, Ugalde JE, Galán JE. Delivery of a *Salmonella* Typhi exotoxin from a host intracellular compartment. Cell Host Microbe 2008; 3:30–8. 18191792 10.1016/j.chom.2007.11.001

[24] Miller RA, Wiedmann M. The cytolethal distending toxin produced by nontyphoidal *Salmonella* serotypes Javiana, Montevideo, Oranienburg, and Mississippi induces DNA damage in a manner similar to that of serotype Typhi. mBio 2016; 7:e02109-16. 10.1128/mBio.02109-16 PMC5181781 27999166

[25] Rodriguez-Rivera LD, Bowen BM, Den Bakker HC, Duhamel GE, Wiedmann M. Characterization of the cytolethal distending toxin (typhoid toxin) in non-typhoidal *Salmonella* serovars. Gut Pathog 2015; 7:19. 26207144 10.1186/s13099-015-0065-1 PMC4511993

[26] Charles RC, Sheikh A, Krastins B, et al Characterization of anti-*Salmonella* enterica serotype typhi antibody responses in bacteremic Bangladeshi patients by an immunoaffinity proteomics-based technology. Clin Vaccine Immunol 2010; 17:1188–95. 20573880 10.1128/CVI.00104-10 PMC2916242

[27] Wallace AJ, Stillman TJ, Atkins A, et al *E. coli* hemolysin E (Hlye, ClyA, SheA): X-ray crystal structure of the toxin and observation of membrane pores by electron microscopy. Cell 2000; 100:265–76. 10660049 10.1016/s0092-8674(00)81564-0

[28] den Bakker HC, Moreno Switt AI, Govoni G, et al Genome sequencing reveals diversification of virulence factor content and possible host adaptation in distinct subpopulations of *Salmonella enterica*. BMC Genomics 2011; 12:425. 21859443 10.1186/1471-2164-12-425 PMC3176500

[29] Oscarsson J, Westermark M, Löfdahl S, et al Characterization of a pore-forming cytotoxin expressed by *Salmonella enterica* serovars Typhi and Paratyphi A. Infect Immun 2002; 70:5759–69. 12228306 10.1128/IAI.70.10.5759-5769.2002 PMC128311

[30] Fuentes JA, Villagra N, Castillo-Ruiz M, Mora GC. The Salmonella Typhi hlyEgene plays a role in invasion of cultured epithelial cells and its functional transfer to *S. Typhimurium* promotes deep organ infection in mice. Res Microbiol 2008; 159:279–87. doi: 10.1016/j.resmic.2008.02.006. Epub 2008 Mar 16. PMID: 18434098. 18434098

[31] Darton TC, Baker S, Randall A, et al Identification of novel serodiagnostic signatures of typhoid fever using a *Salmonella* proteome array. Front Microbiol 2017; 8:1794. doi: 10.3389/fmicb.2017.01794. PMID: 28970824; PMCID: PMC5609549. 28970824 PMC5609549

[32] Andrews JR, Khanam F, Rahman N, et al Plasma immunoglobulin A responses against 2 *Salmonella* Typhi antigens identify patients with typhoid fever. Clin Infect Dis 2019; 68:949–55. 30020426 10.1093/cid/ciy578 PMC6399438

[33] Kumar S, Nodoushani A, Khanam F, et al Evaluation of a rapid point-of-care multiplex immunochromatographic assay for the diagnosis of enteric fever. mSphere 2020; 5:e00253-20. 32522777 10.1128/mSphere.00253-20 PMC7289704

[34] Blohmke CJ, Muller J, Gibani MM, et al Diagnostic host gene signature for distinguishing enteric fever from other febrile diseases. EMBO Mol Med 2019; 11:e10431 doi: 10.15252/emmm.201910431. Epub 2019 Aug 30. PMID: 31468702; PMCID: PMC6783646. 31468702 PMC6783646

