# Supplementary material for: The Identification of Enteric Fever-Specific Antigens for Population-Based Serosurveillance
Source: J Infect Dis. 2023 Jul 5;229(3):833–44. doi: 10.1093/infdis/jiad242 (PMC10938218; doi:10.1093/infdis/jiad242)
Supplement: jiad242_Supplementary_Data [file jiad242_supplementary_data.docx]

**The identification of enteric fever-specific antigens for population based serosurveillance**

Elli Mylona ^1,2,†^, Lisa Hefele ^3,†^, Nga Tran Vu Thieu ^4, †^, Tan Trinh Van ^4^, Chau Nguyen Ngoc Minh ^4^,

Anh Tran Tuan ^4^, Abhilasha Karkey ^5^, Sabina Dongol ^5^, Buddha Basnyat ^5^, Phat Voong Vinh ^4^,

Thanh Ho Ngoc Dan ^4^, Paula Russell ^1,2^, Richelle C. Charles ^7^, Christopher M Parry ^6,8^, and Stephen Baker ^1,2,*^

^1^ Cambridge Institute of Therapeutic Immunology and Infectious Disease, University of Cambridge School of Clinical Medicine, Cambridge Biomedical Campus, Cambridge, United Kingdom

^2^ Department of Medicine, University of Cambridge School of Clinical Medicine, Cambridge Biomedical Campus, Cambridge, United Kingdom

^3^ Department of Infection and Immunity, Luxembourg Institute of Health, Esch-sur-Alzette, Grand-Duchy of Luxembourg

^4^ The Hospital for Tropical Diseases, Wellcome Trust Major Overseas Programme, Oxford University Clinical Research Unit, Ho Chi Minh City, Vietnam

^5^ Oxford University Clinical Research Unit, Patan Academy of Health Sciences, Kathmandu, Nepal

^6^ Centre for Tropical Medicine, Oxford University, Oxford, United Kingdom

^7^ Harvard Medical School, Massachusetts General Hospital, Boston, USA

^8^ Clinical Sciences, Liverpool School of Tropical Medicine, Liverpool, United Kingdom

^†^ Contributed equally

^*^ Corresponding author: Prof. Stephen Baker, Cambridge Institute of Therapeutic Immunology and Infectious Disease, The Department of Medicine, University of Cambridge, Cambridge, UK

# Data analysis

Data analyses were conducted in R software [1] with the following packages: “stats” [1], “tydiverse” [2], “gtsummary” [3], “ggpubr” [4], “rstatix” [5], “cowplot” [6], “ggbeeswarm” [7], “ggcorrplot” [8] “GGally”[9] and “janitor” [10].

# Tables

Table S1. The number of missing samples per time point

| **Patient group** |  | **Day 1** | **Day 8** | **Month 1** | **Month 3** |
| --- | --- | --- | --- | --- | --- |
| **ST** | O2 | 1/103 (0.97) | 1/103 (0.97) | 2/103 (1.94) | 62/103 (60.19) |
|  | O9 | 2/103 (1.94) | 1/103 (0.97) | 3/103 (2.91) | 63/103 (61.17) |
|  | STY0452 (yajl) | 1/103 (0.97) | 1/103 (0.97) | 2/103 (1.94) | 62/103 (60.19) |
|  | STY0796 (ybgF) | 1/103 (0.97) | 1/103 (0.97) | 2/103 (1.94) | 62/103 (60.19) |
|  | STY1086 | 1/103 (0.97) | 1/103 (0.97) | 2/103 (1.94) | 62/103 (60.19) |
|  | STY1372 (pspB) | 1/103 (0.97) | 1/103 (0.97) | 2/103 (1.94) | 62/103 (60.19) |
|  | STY1479 (yncE) | 1/103 (0.97) | 1/103 (0.97) | 2/103 (1.94) | 62/103 (60.19) |
|  | STY1498 (hlyE) | 2/103 (1.94) | 1/103 (0.97) | 3/103 (2.91) | 63/103 (61.17) |
|  | STY1522 | 1/103 (0.97) | 1/103 (0.97) | 2/103 (1.94) | 62/103 (60.19) |
|  | STY1612 | 1/103 (0.97) | 1/103 (0.97) | 2/103 (1.94) | 62/103 (60.19) |
|  | STY1703 (ssaP) | 1/103 (0.97) | 1/103 (0.97) | 2/103 (1.94) | 62/103 (60.19) |
|  | STY1767 (nlpC) | 1/103 (0.97) | 1/103 (0.97) | 2/103 (1.94) | 62/103 (60.19) |
|  | STY1886 (cdtB) | 1/103 (0.97) | 1/103 (0.97) | 2/103 (1.94) | 62/103 (60.19) |
|  | STY3208 | 1/103 (0.97) | 1/103 (0.97) | 2/103 (1.94) | 62/103 (60.19) |
|  | STY4190 (yhjJ) | 1/103 (0.97) | 1/103 (0.97) | 2/103 (1.94) | 62/103 (60.19) |
|  | STY4539 (piIL) | 1/103 (0.97) | 1/103 (0.97) | 2/103 (1.94) | 62/103 (60.19) |
|  | Vi | 1/103 (0.97) | 1/103 (0.97) | 2/103 (1.94) | 62/103 (60.19) |
| **SPA** | O2 | 3/61 (4.92) | 0/61 (0) | 0/61 (0) | 15/61 (24.59) |
|  | O9 | 3/61 (4.92) | 1/61 (1.64) | 1/61 (1.64) | 18/61 (29.51) |
|  | STY0452 (yajl) | 3/61 (4.92) | 0/61 (0) | 0/61 (0) | 15/61 (24.59) |
|  | STY0796 (ybgF) | 3/61 (4.92) | 0/61 (0) | 0/61 (0) | 15/61 (24.59) |
|  | STY1086 | 3/61 (4.92) | 0/61 (0) | 0/61 (0) | 15/61 (24.59) |
|  | STY1372 (pspB) | 3/61 (4.92) | 0/61 (0) | 0/61 (0) | 15/61 (24.59) |
|  | STY1479 (yncE) | 3/61 (4.92) | 0/61 (0) | 0/61 (0) | 15/61 (24.59) |
|  | STY1498 (hlyE) | 3/61 (4.92) | 1/61 (1.64) | 1/61 (1.64) | 18/61 (29.51) |
|  | STY1522 | 3/61 (4.92) | 0/61 (0) | 0/61 (0) | 15/61 (24.59) |
|  | STY1612 | 3/61 (4.92) | 0/61 (0) | 0/61 (0) | 15/61 (24.59) |
|  | STY1703 (ssaP) | 3/61 (4.92) | 0/61 (0) | 0/61 (0) | 15/61 (24.59) |
|  | STY1767 (nlpC) | 3/61 (4.92) | 0/61 (0) | 0/61 (0) | 15/61 (24.59) |
|  | STY1886 (cdtB) | 3/61 (4.92) | 0/61 (0) | 0/61 (0) | 15/61 (24.59) |
|  | STY3208 | 3/61 (4.92) | 0/61 (0) | 0/61 (0) | 15/61 (24.59) |
|  | STY4190 (yhjJ) | 3/61 (4.92) | 0/61 (0) | 0/61 (0) | 15/61 (24.59) |
|  | STY4539 (piIL) | 3/61 (4.92) | 0/61 (0) | 0/61 (0) | 15/61 (24.59) |
|  | Vi | 3/61 (4.92) | 0/61 (0) | 0/61 (0) | 15/61 (24.59) |
| **FCN** | O2 | 2/322 (0.62) | 0/322 (0) | 51/322 (15.84) | 139/322 (43.17) |
|  | O9 | 2/322 (0.62) | 1/322 (0.31) | 53/322 (16.46) | 142/322 (44.1) |
|  | STY0452 (yajl) | 2/322 (0.62) | 0/322 (0) | 51/322 (15.84) | 139/322 (43.17) |
|  | STY0796 (ybgF) | 2/322 (0.62) | 0/322 (0) | 51/322 (15.84) | 139/322 (43.17) |
|  | STY1086 | 2/322 (0.62) | 0/322 (0) | 51/322 (15.84) | 139/322 (43.17) |
|  | STY1372 (pspB) | 2/322 (0.62) | 0/322 (0) | 51/322 (15.84) | 139/322 (43.17) |
|  | STY1479 (yncE) | 2/322 (0.62) | 0/322 (0) | 51/322 (15.84) | 139/322 (43.17) |
|  | STY1498 (hlyE) | 2/322 (0.62) | 1/322 (0.31) | 53/322 (16.46) | 142/322 (44.1) |
|  | STY1522 | 2/322 (0.62) | 0/322 (0) | 51/322 (15.84) | 139/322 (43.17) |
|  | STY1612 | 2/322 (0.62) | 0/322 (0) | 51/322 (15.84) | 139/322 (43.17) |
|  | STY1703 (ssaP) | 2/322 (0.62) | 0/322 (0) | 51/322 (15.84) | 139/322 (43.17) |
|  | STY1767 (nlpC) | 2/322 (0.62) | 0/322 (0) | 51/322 (15.84) | 139/322 (43.17) |
|  | STY1886 (cdtB) | 2/322 (0.62) | 0/322 (0) | 51/322 (15.84) | 139/322 (43.17) |
|  | STY3208 | 2/322 (0.62) | 0/322 (0) | 51/322 (15.84) | 139/322 (43.17) |
|  | STY4190 (yhjJ) | 2/322 (0.62) | 0/322 (0) | 51/322 (15.84) | 139/322 (43.17) |
|  | STY4539 (piIL) | 2/322 (0.62) | 0/322 (0) | 51/322 (15.84) | 139/322 (43.17) |
|  | Vi | 2/322 (0.62) | 0/322 (0) | 51/322 (15.84) | 139/322 (43.17) |
| FCN = febrile, culture-negative patients; SPA = S. Paratyphi A-confirmed patients; ST = S. Typhi-confirmed patients | | | | | |

Table S2. Plasmid constructs of *S*. Typhi antigens generated for protein expression and antigen purification

| Target | Construct name | 5'RE | Ty2 nomenclature and Coding sequence | 3'RE | Forward primer | Reverse primer | bp | aa | kDa |
| --- | --- | --- | --- | --- | --- | --- | --- | --- | --- |
| STY4190 | pEK90 | NcoI | 3904 (Typhi) aa25-495 | NotI | catg ccatgg gt  GATGCGCTCCAGCCCGATC | gcatgagc gcggccgc  CTGTGCCGCCGGTGTTTCC | 1413 | 471 | 52.6 |
| STY3208 | pEK91 | NcoI | 2970 (Typhi) aa1-279 | NotI | catg ccatgg gt  ATGGCAGCTAACGGAGAAAATAATCC | gcatgagc gcggccgc  CCAGGTCTTACCTATTTTAAATTCCC | 837 | 279 | 30.5 |
| STY1767 | pEK92 | NcoI | 1224 (Typhi) aa21-154 | NotI | catg ccatgg gt  GCACCGGCGCCAAATGCCAG | gcatgagc gcggccgc  AATTCGCCGTGCCTGCCAGAAG | 402 | 134 | 15.1 |
| STY1703 | pEK93 | NcoI | 1285 (Typhi) aa1-124 | NotI | catg ccatgg gt  ATGCGTATTACCAAAGTTGAGGG | gcatgagc gcggccgc TTCGCTATTCTTAACATAGAATATCTC | 372 | 124 | 14.1 |
| STY1522 | pEK94 | NcoI | 1459 (Typhi) aa25-363 | NotI | catg ccatgg gt TGCACAACCCTTGCTATTCAGGATAAAC | gcatgagc gcggccgc TCCTTTGACGTTGATTTTCTCGAACAC | 1017 | 339 | 37.6 |
| STY1886 | pEK95 | NcoI | 1111 (Typhi) aa28-269 | NotI | catg ccatgg gt  AAAGTTATGACCTGGAATCTTCAGGG | gcatgagc gcggccgc  ACAGCTTCGTGCCAAAAAGGCTAC | 726 | 242 | 26.4 |
| STY1498 | pEK96 | NcoI | 1477 (Typhi) aa203-305 | NotI | catg ccatgg gt  GGCGTGATTGAAGGGAAATTGATTCc | gcatgagc gcggccgc  GACGTCAGGAACCTCGAAAAGCG | 309 | 103 | 11.5 |
| STY3375 | pEK99 | NcoI | 3116 (Typhi) aa1-118 | NotI | catg ccatgg gt  ATGGCGTCCACATATCGCAC | gcatgagc gcggccgc  CTCTTGTGGATCGACTGGC | 354 | 118 | 13.8 |
| STY1372 | pEK100 | NcoI | 1594 (Typhi) aa27-74 | NotI | catg ccatgg gt  AGCAACCGCGCCGGTCGG | gcatgagc gcggccgc  GCGCTCTCTCCAGTTCGGATG | 144 | 48 | 5.7 |
| STY1612 | pEK101 | NcoI | 1376 (Typhi) aa30-108 | NotI | catg ccatgg gt  AGTAAAACAGAAGAACGCCAGGC | gcatgagc gcggccgc  TTGACCTCCGGTATTGCGGTAC | 237 | 79 | 9.2 |
| STY0357 | pEK102 | NcoI | 2538 (Typhi) aa20-246 | NotI | catg ccatgg gt  GGTTTGCTGAGCAGCAGCAGC | gcatgagc gcggccgc  TTTTGCCTCGGAGAGCGTATAATTTG | 681 | 227 | 25.8 |
| STY4539 | pEK103 | NcoI | 4239 (Typhi) aa20-414 | NotI | catg ccatgg gt  CAGCAGACCTCCACCCAAACC | gcatgagc gcggccgc  CGACCGGTCAGCCGGTTTATC | 1185 | 395 | 41.9 |
| STY0452 | pEK104 | NcoI | 2449 (Typhi) aa22-179 | NotI | catg ccatgg gt  CCGCAGAGCGAAGTTCGC | gcatgagc gcggccgc  TTGATTAACAGGCTGAATATCATGG | 474 | 158 | 17.2 |
| STY0796 | pEK105 | NcoI | 2126 (Typhi) aa27-262 | NotI | catg ccatgg gt  CAGGCGCCAATCAGTAGTGTC | gcatgagc gcggccgc  CATCGCGTTAAGACGCTTCTGC | 708 | 236 | 25.4 |
| STY0065 | pEK106 | NcoI | 0058 (Typhi) aa33-79 | NotI | catg ccatgg gt  GGTATGTCTGCCGTGATTACTC | gcatgagc gcggccgc  AGCGTTAAGGCGGTGATGGTG | 138 | 46 | 4.9 |
| STY3093 | pEK107 | NcoI | 2864 (Typhi) aa1-118 | NotI | catg ccatgg gt  ATGTTTTTGACGTACATTTCATTTCagg | gcatgagc gcggccgc  CTGGCCTTTGGCGTTAATTTTAC | 354 | 118 | 13.6 |
| STY1086 | pEK108 | PciI | 1855 (Typhi) aa20-178 | NotI | catg acatgt ta  GGAGAAAATAAAAGCTATTATCAGCTC | gcatgagc gcggccgc  AGCGGCCGCTTCCTGACTC | 477 | 159 | 17.4 |
| STY3765 | pEK109 | NcoI | 3515 (Typhi) aa1-518 | NotI | catg ccatgg gt  ATGAAAGTAAAACTGCTTGCTGCC | gcatgagc gcggccgc  CTTCTTCACATCCGCAACACG | 1554 | 518 | 55.5 |

Table S3. Salmonella Typhi/Salmonella Paratyphi A Antigens Used in This Study for Serological Testing

| **#** | **Ag** | **Gene name** | **Annotation** |
| --- | --- | --- | --- |
| 1 | STY1372 | pspB | Phage shock protein B |
| 2 | STY1612 |  | Putative membrane protein – prophage associated |
| 3 | STY4539 | pilL | Putative exported protein – type IV pili |
| 4 | STY0452 | yajI | Putative lipoprotein – Prokaryotic homolog of protein DJ-1 |
| 5 | STY0796 | ybgF | Putative exported protein – Tol/pal system protein |
| 6 | STY1086 |  | Putative lipoprotein |
| 7 | STY4190 | yhjJ | Putative Zinc-protease |
| 8 | STY3208 |  | Hypothetical protein – Unknown function |
| 9 | STY1767 | nlpC | Putative lipoprotein – Endopeptidase |
| 10 | STY1498 | hlyE | hemolysin |
| 11 | STY1703 | ssaP | Putative secreted protein – T3SS |
| 12 | STY1522 |  | Putative secreted protein – Choloylglycine secreted homolog |
| 13 | STY1886 | cdtB | Cytolethal distending toxin subunit B homolog |
| 14 | STY1479 | yncE | Possible ATP-binding protein |
| 15 | Vi |  | virulence antigen |
| 16 | O2 |  | O antigens (SPA) (somatic, lipopolysaccharide) |
| 17 | O9 |  | O antigens (ST) (somatic, lipopolysaccharide) |

# Figures


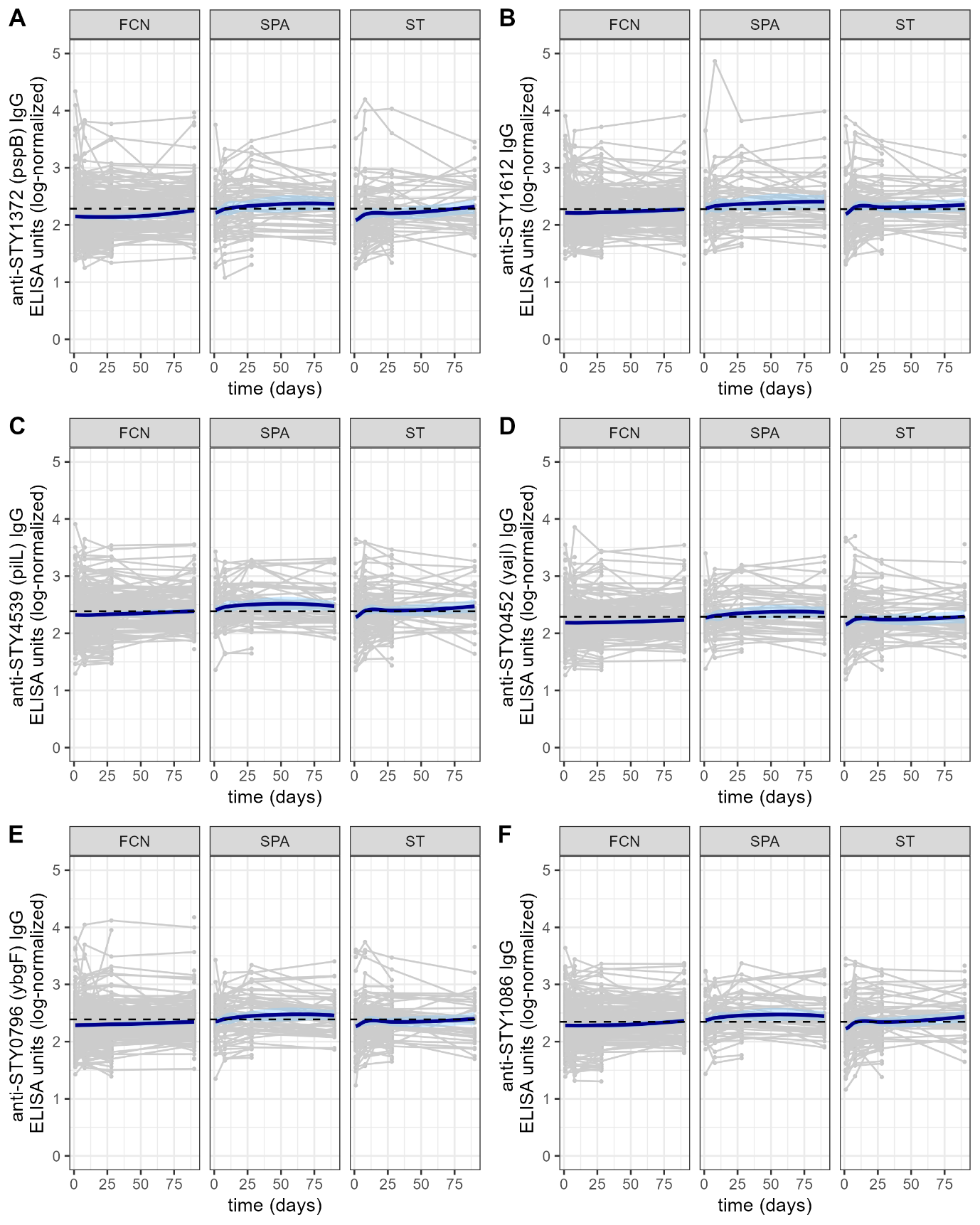


Figure S1. The longitudinal IgG responses against *S*. Typhi or *S*. Paratyphi A purified antigens in a Nepali cohort of febrile patients.

IgG antibody responses against STY1372 (A), STY1612 (B), STY4539 (C), STY0452 (D), STY0796 (E) and STY1086 (F) over the course of three months in different patient groups. FCN, culture negative febrile patients, SPA, *S*. Paratyphi A confirmed patients, ST, *S*. Typhi-confirmed patients. Grey lines show antibody trajectories in individual patients and the blue line the fitted loess smooth function. Dashed lines represent the mean titre value within the control group of afebrile, community samples for the respective antibody.


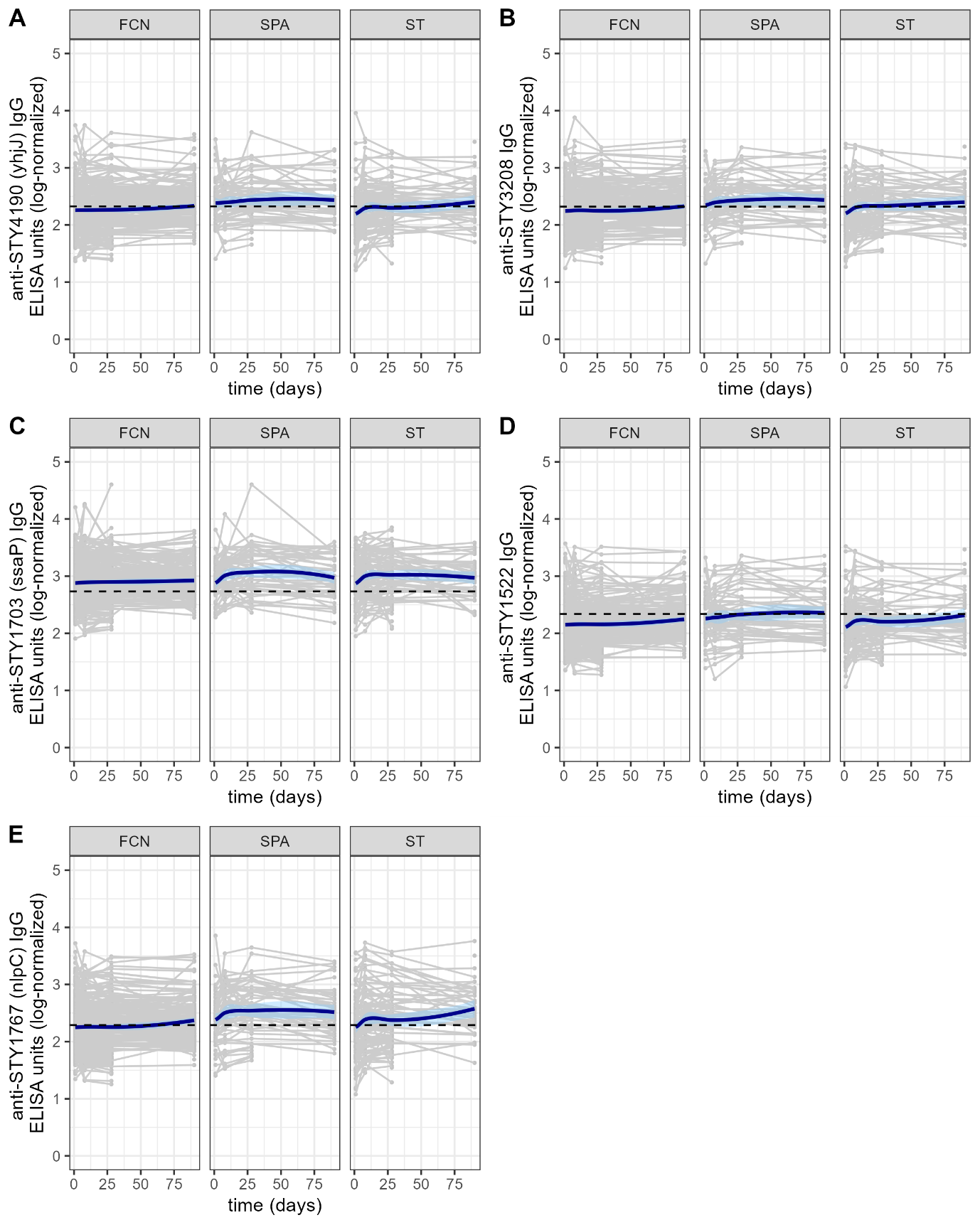
Figure S2. The longitudinal IgG responses against *S*. Typhi or *S*. Paratyphi A antigens in a Nepali cohort of febrile patients.

IgG antibody responses against STY4190 (A), STY3208 (B), STY1703 (C), STY1522 (D) and STY1767 (E) over the course of three months in different patient groups. FCN, culture negative febrile patients, SPA, *S*. Paratyphi A confirmed patients, ST, *S*. Typhi-confirmed patients. Grey lines show antibody trajectories in individual patients and the blue line the fitted loess smooth function. Dashed lines represent the mean titre value within the control group of afebrile, community samples for the respective antibody.


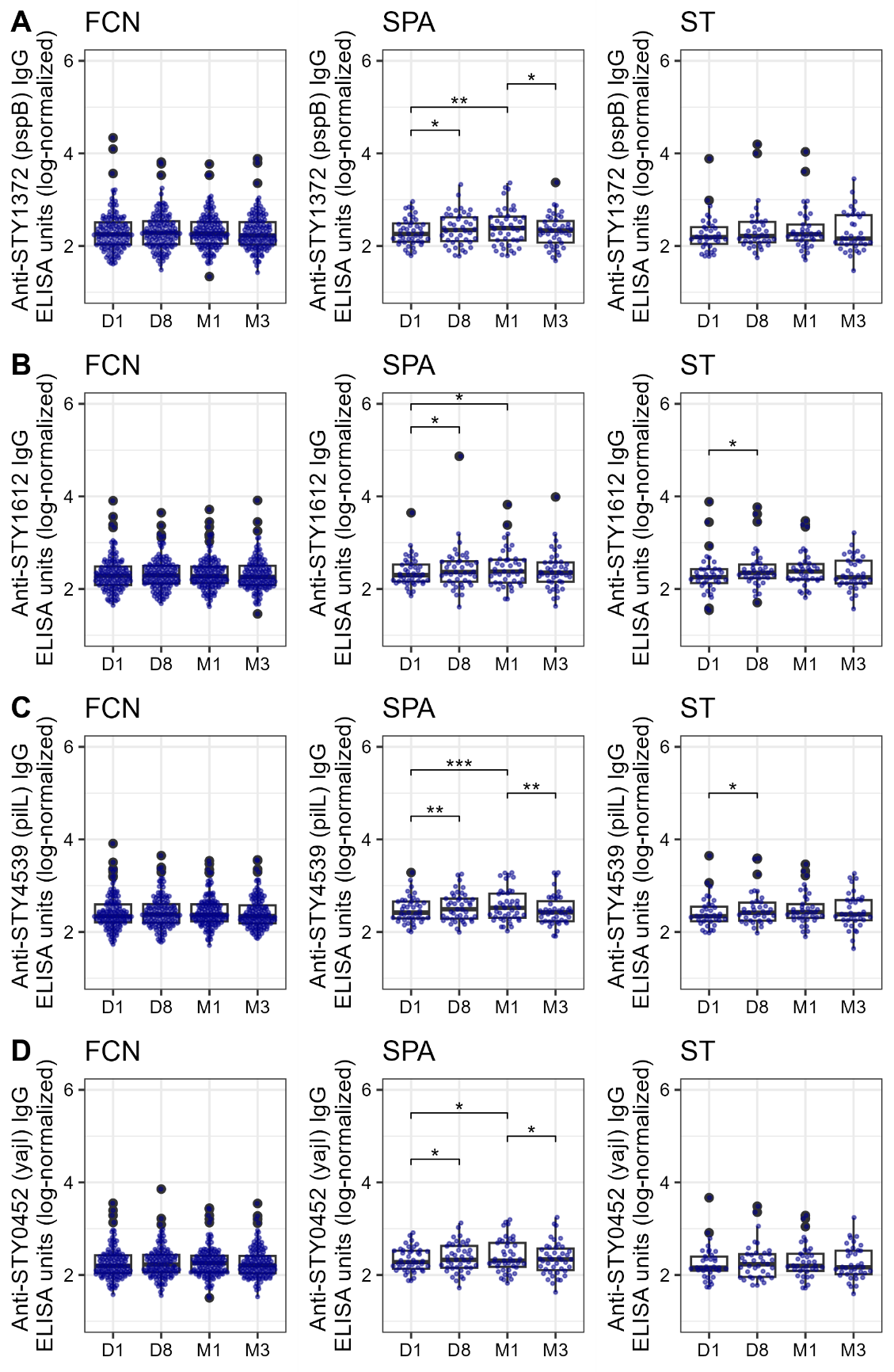


Figure S3. The distribution of serum IgG titres in a Nepali cohort of enteric fever patients and febrile culture-negative controls.

Boxplots showing IgG levels in plasma from febrile, culture-negative patients (FCN), *S*. Paratyphi A (SPA) or *S*. Typhi (ST) against STY1372 (A), STY1612 (B), STY4539 (C) and STY0452 (D) antigens over the course of three months. Each dot shows the antibody titer of an individual sample on day 1 (D1), day 8 (D8), day 28 (M1) and day 90 (M3). Differences between time points were assessed using Friedman test followed by pairwise comparisons using Wilcoxon signed-rank tests. P-values were adjusted using the Bonferroni multiple testing correction method. *p<0.05, **p<0.01, ***p<0.001, ****p<0.0001.


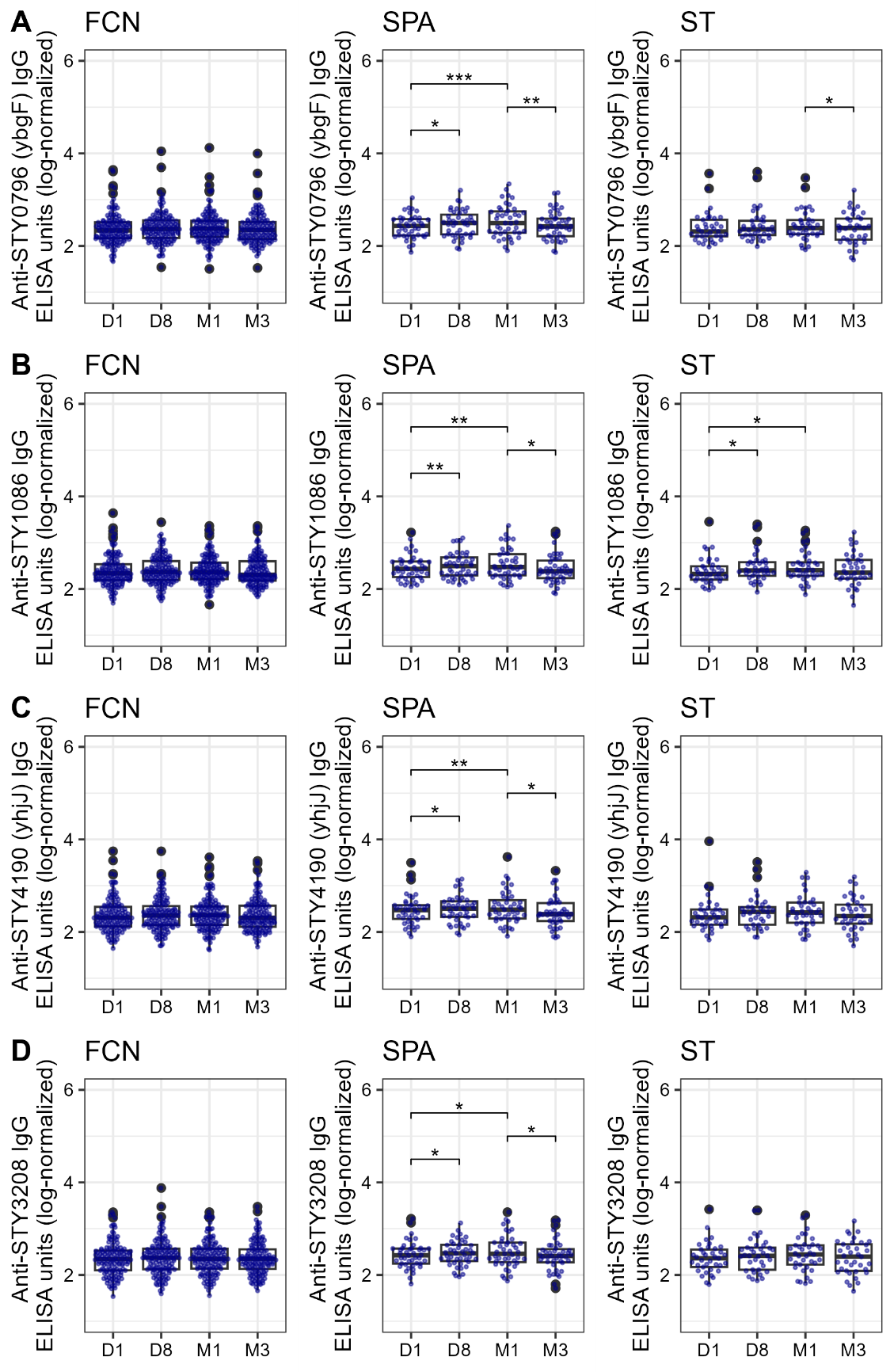


Figure S4. The distribution of serum IgG titers in a Nepali cohort of of enteric fever patients and febrile culture-negative controls.

Boxplots showing IgG levels in plasma from febrile, culture-negative patients (FCN), *S*. Paratyphi A (SPA) or *S*. Typhi (ST) against STY0796 (A), STY1086 (B), STY4190 (C) and STY3208 (D) antigens over the course of three months. Each dot shows the antibody titer of an individual sample on day 1 (D1), day 8 (D8), day 28 (M1) and day 90 (M3). Differences between time points were assessed using Friedman test followed by pairwise comparisons using Wilcoxon signed-rank tests. P-values were adjusted using the Bonferroni multiple testing correction method. *p<0.05, **p<0.01, ***p<0.001, ****p<0.0001.


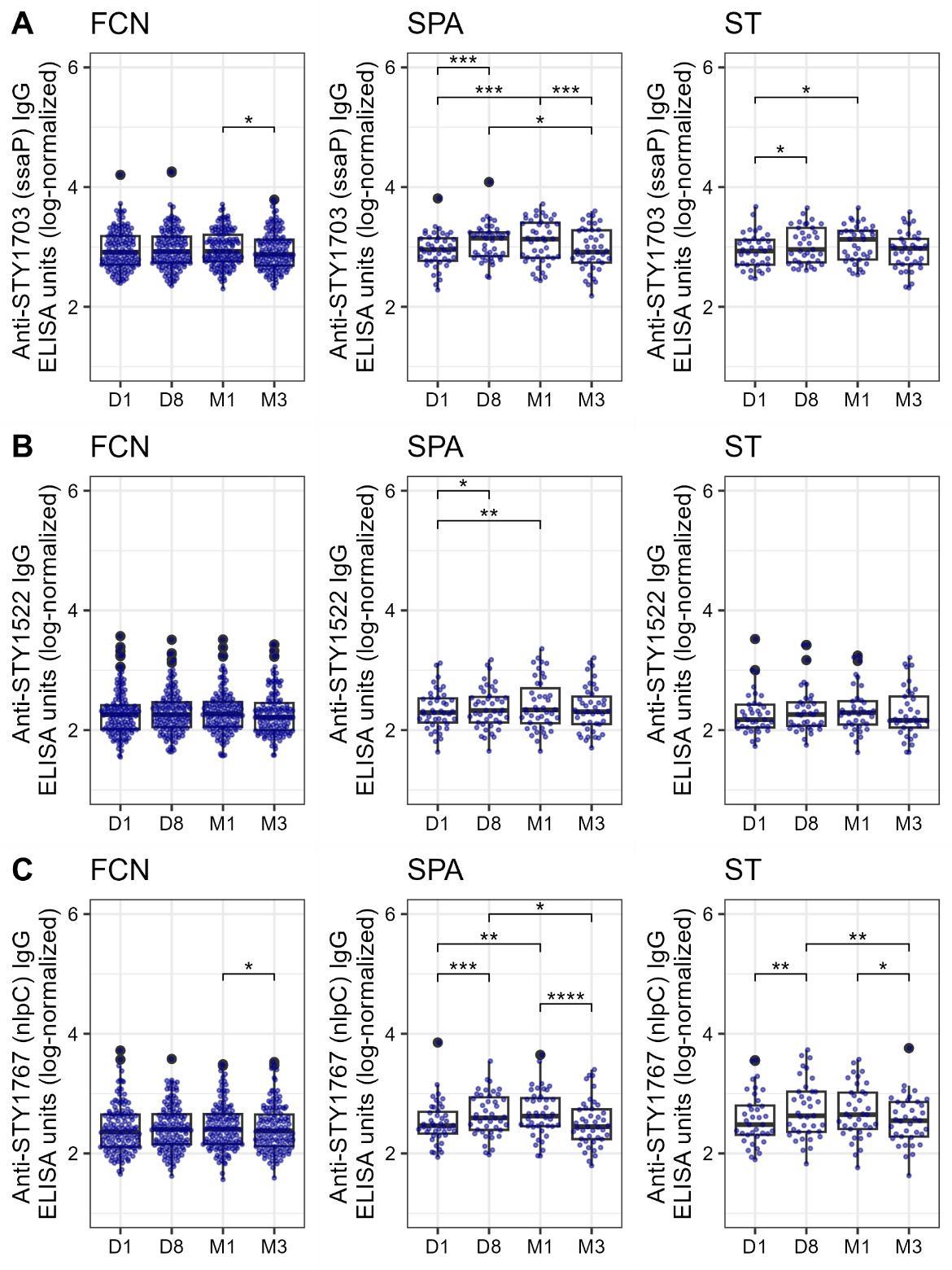


Figure S5. The distribution of serum IgG titers in a Nepali cohort of of enteric fever patients and febrile culture-negative controls.

Boxplots showing IgG levels in plasma from febrile, culture-negative patients (FCN), *S*. Paratyphi A (SPA) or *S*. Typhi (ST) against STY1703 (A), STY1522 (B), and STY1767 (C) antigens over the course of three months. Each dot shows the antibody titer of an individual sample on day 1 (D1), day 8 (D8), day 28 (M1) and day 90 (M3). Differences between time points were assessed using Friedman test followed by pairwise comparisons using Wilcoxon signed-rank tests. P-values were adjusted using the Bonferroni multiple testing correction method. *p<0.05, **p<0.01, ***p<0.001, ****p<0.0001.


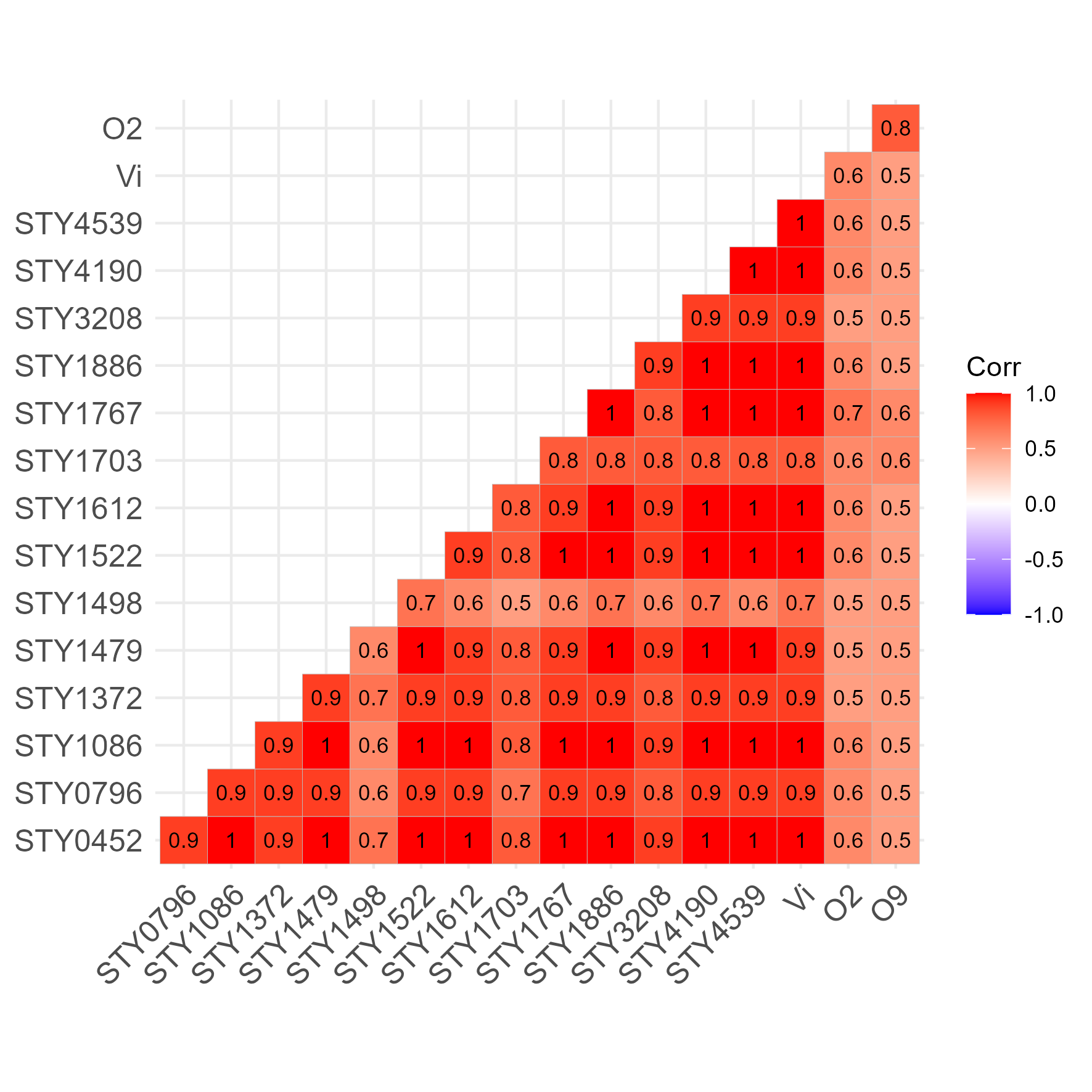


Figure S6. Spearman correlation coefficient (rho) values between antibody responses to the complete panel of *Salmonella* antigens in the group of afebrile controls on day 8.


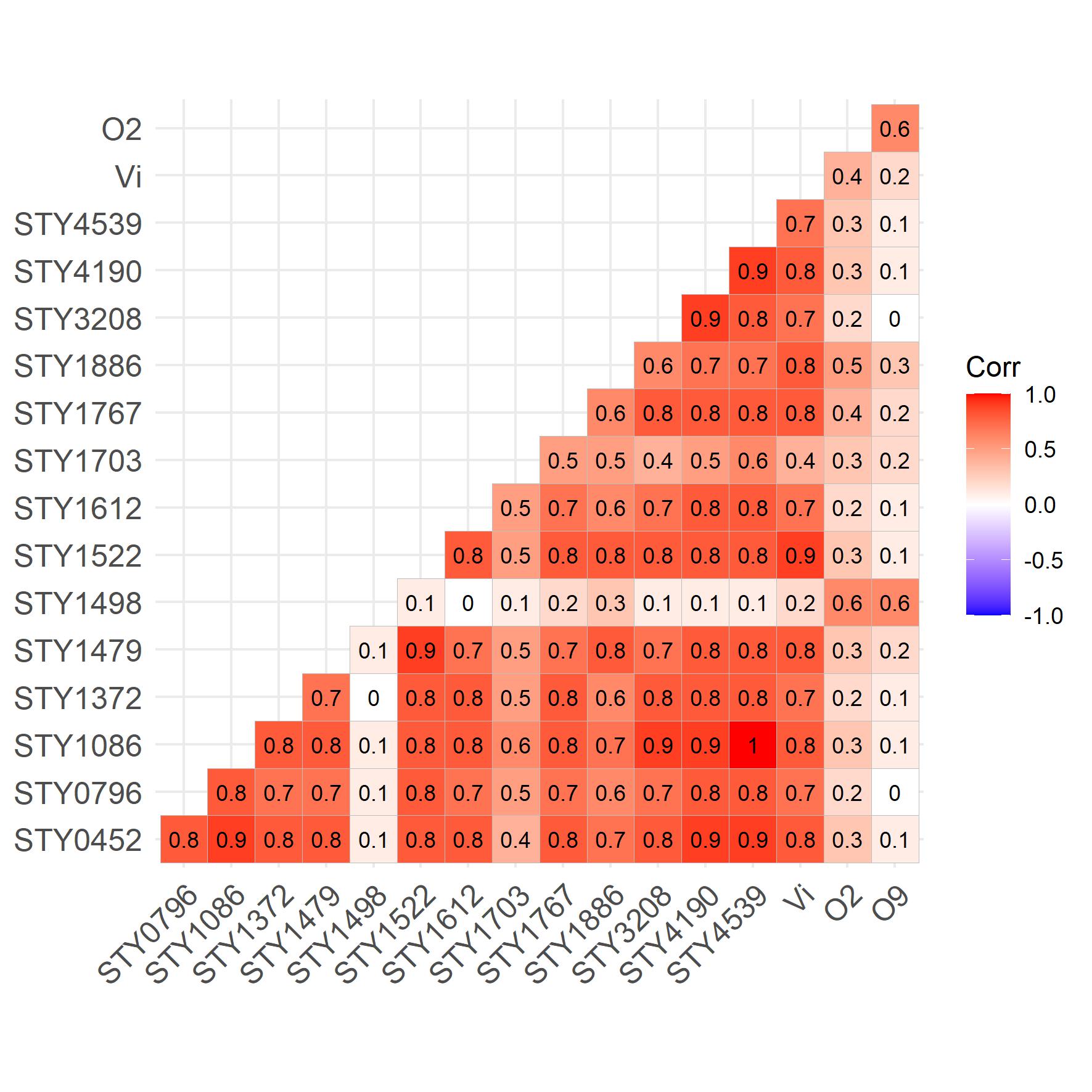


Figure S7. Spearman correlation coefficient (rho) values between antibody responses to the complete panel of *Salmonella* antigens in the group of febrile, culture-negative patients on day 8.


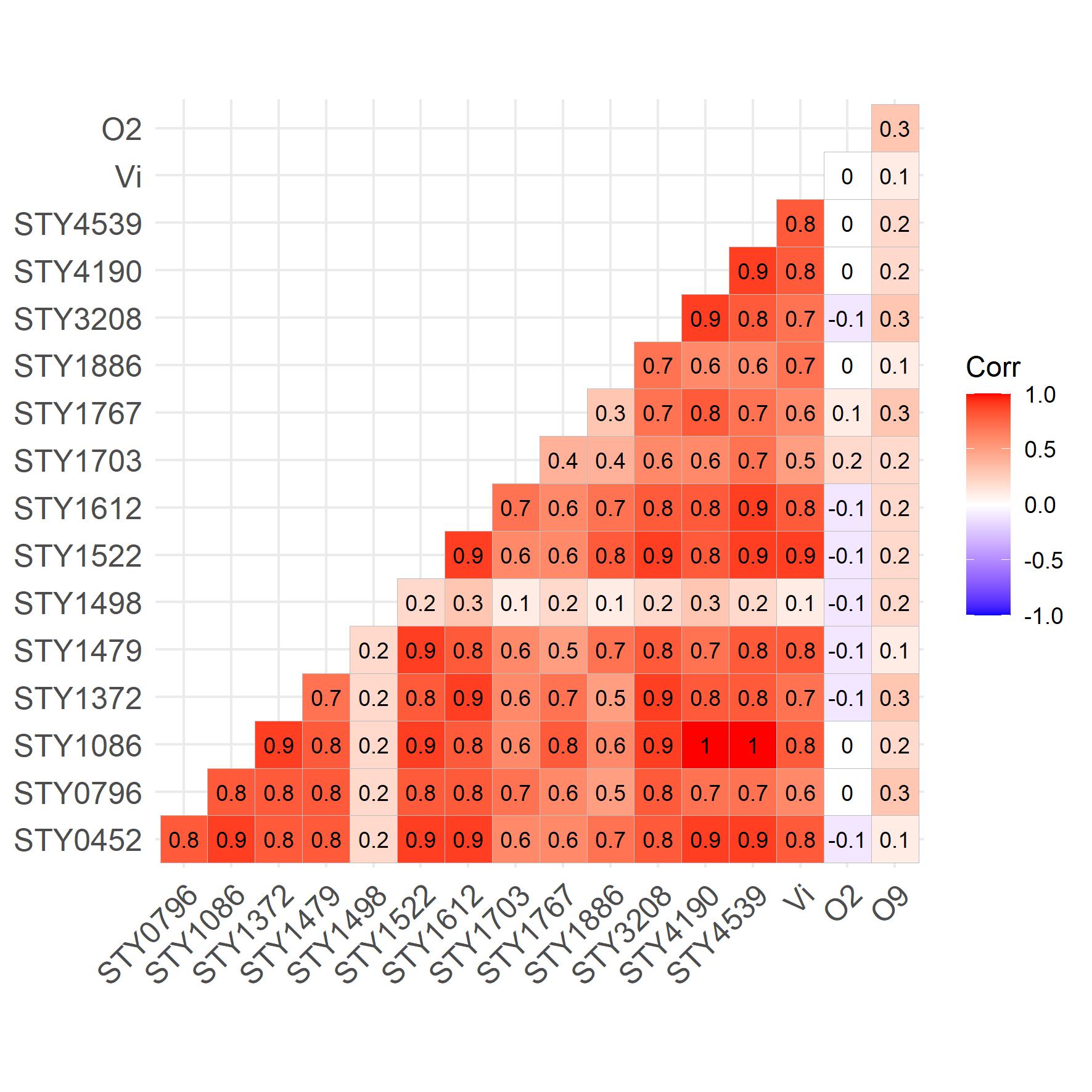


Figure S8. Spearman correlation coefficient (rho) values between antibody responses to the complete panel of *Salmonella* antigens in the group of patients infected with *S*. Paratyphi A on day 8.


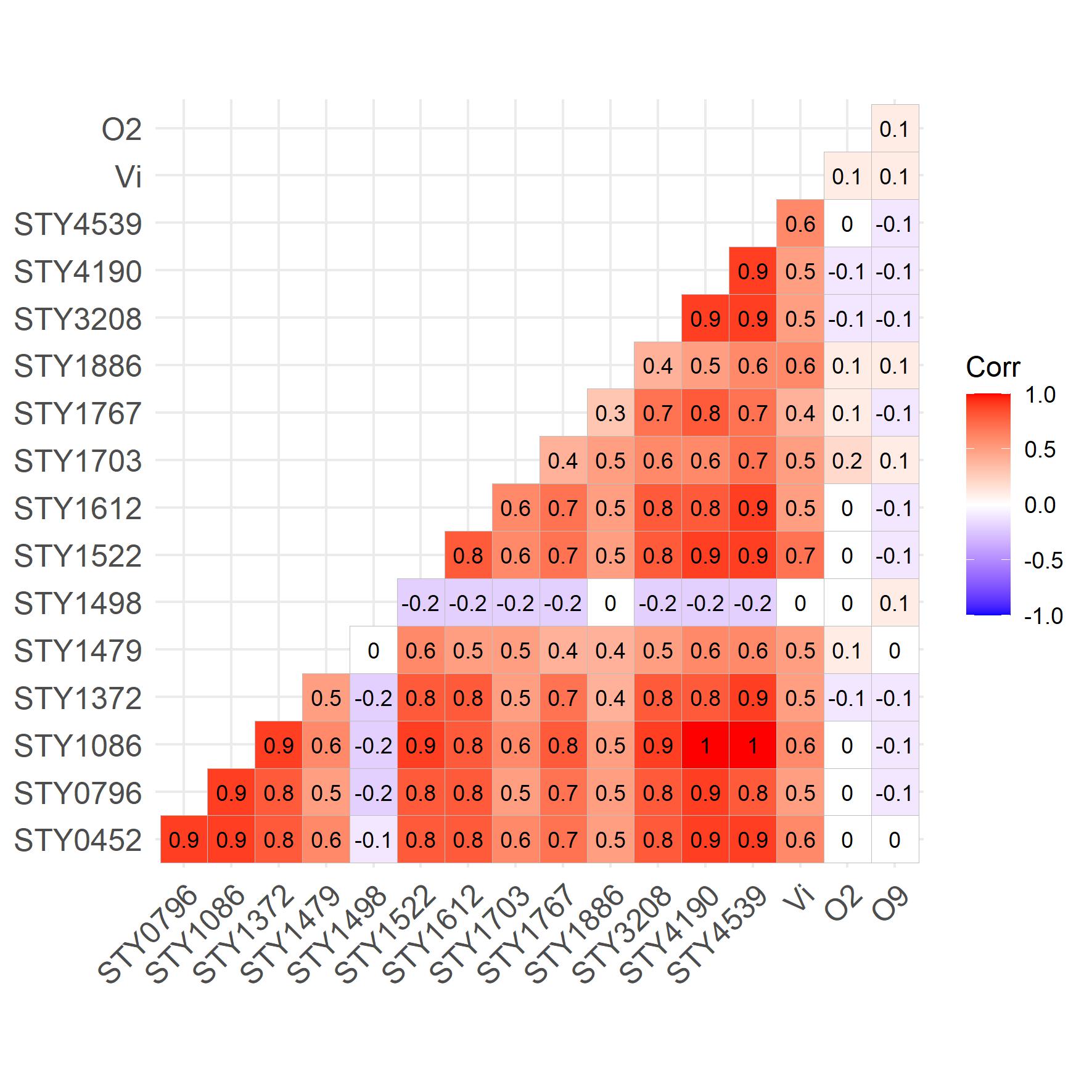


Figure S9. Spearman correlation coefficient (rho) values between antibody responses to the complete panel of *Salmonella* antigens in the group of patients infected with *S*. Typhi on day 8.


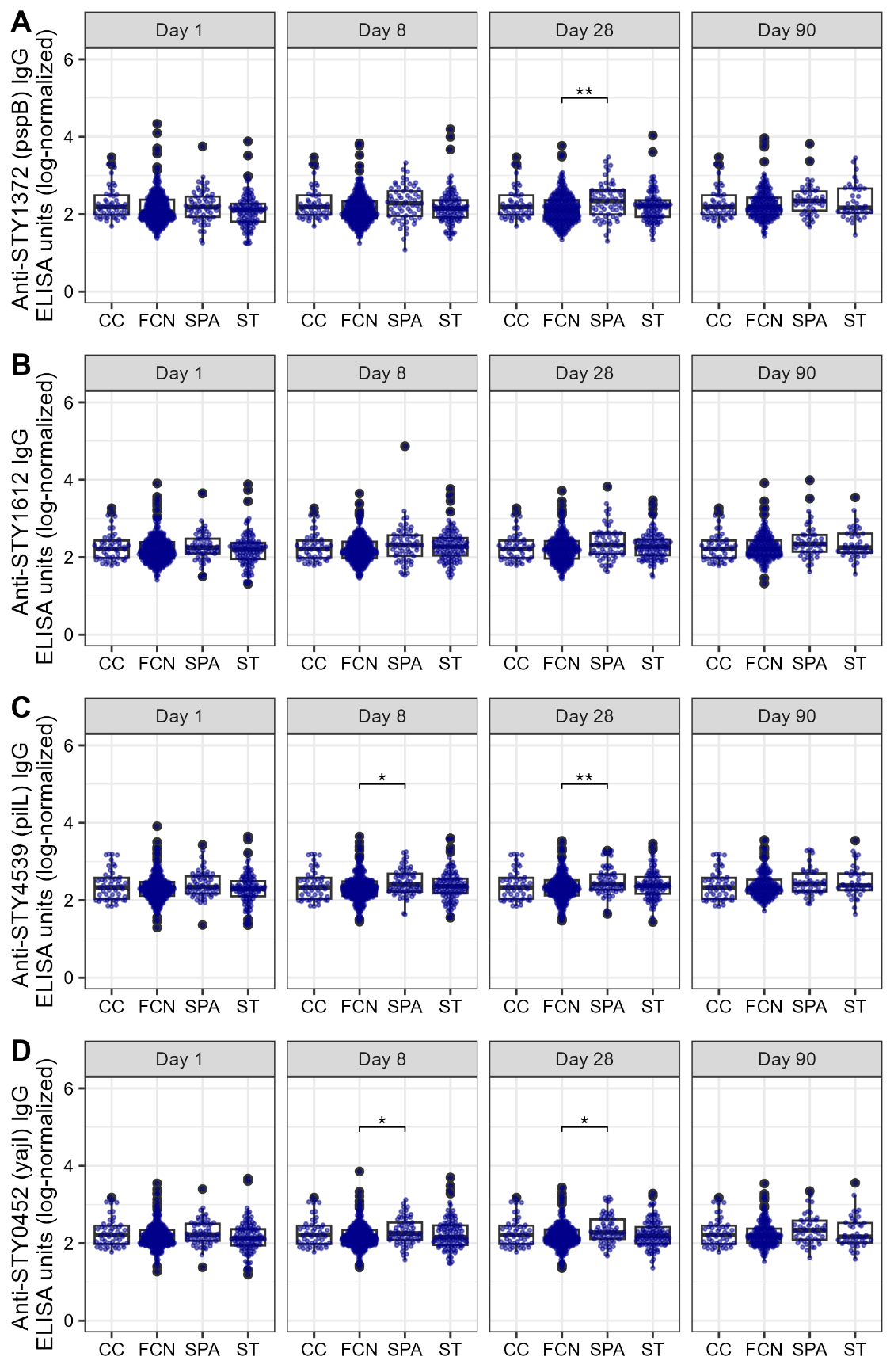


Figure S10. The distribution of serum IgG titers in a Nepali cohort of febrile patients and controls.

Boxplots showing IgG levels in plasma from controls (Ctrl), febrile, culture-negative patients (FCN), *S*. Paratyphi A (SPA) or *S*. Typhi (ST) against STY1372 (A), STY1612 (B), STY4539 (C) and STY0452 (D) antigens over the course of three months. Differences between time points were assessed using Kruskal-Wallis test followed by pairwise comparisons using Wilcoxon signed-rank tests. P-values were adjusted using the Bonferroni multiple testing correction method. *p<0.05, **p<0.01, ***p<0.001, ****p<0.0001.


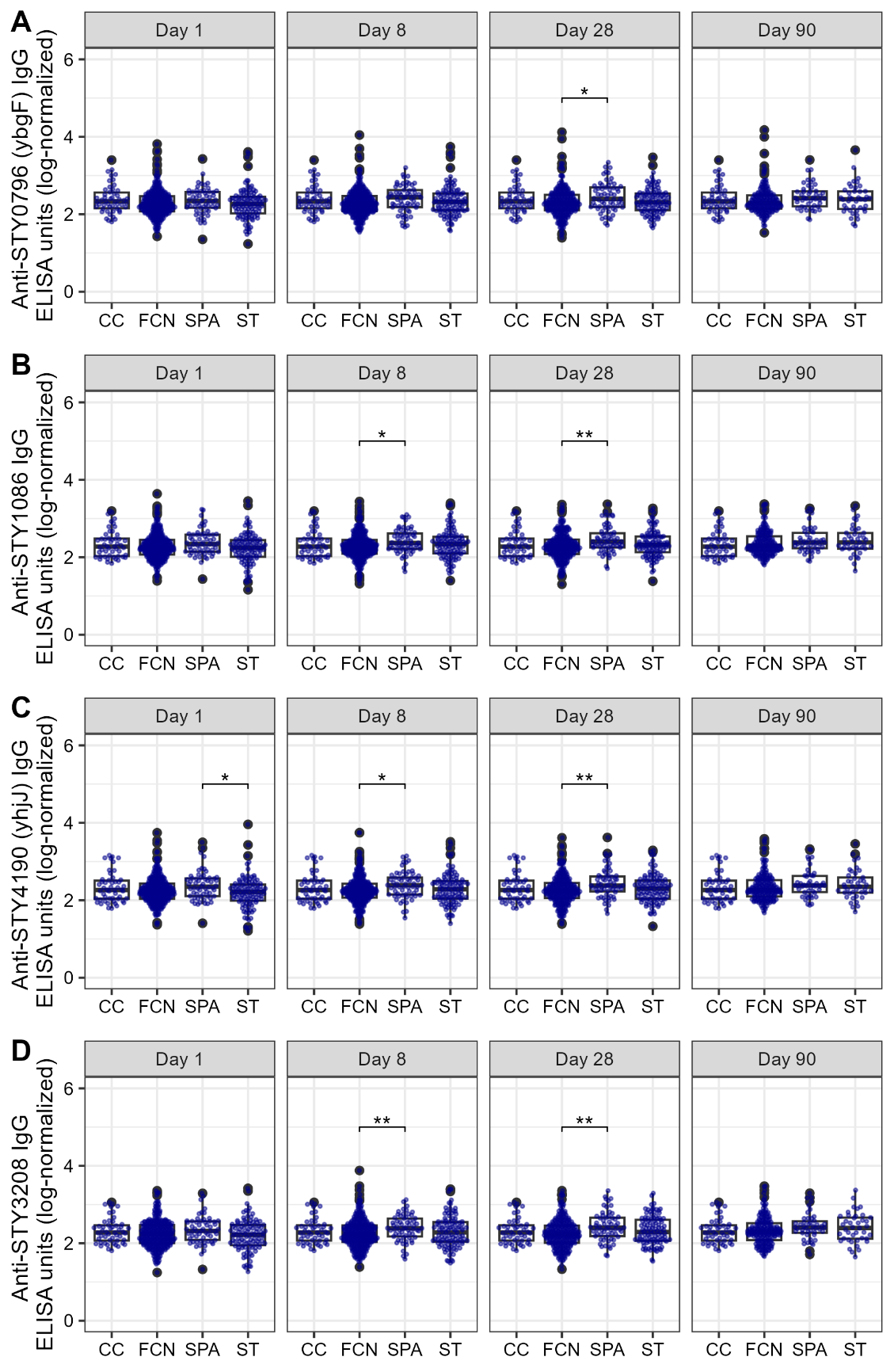


Figure S11. The distribution of serum IgG titers in a Nepali cohort of febrile patients and controls.

Boxplots showing IgG levels in plasma from controls (Ctrl), febrile, culture-negative patients (FCN), *S*. Paratyphi A (SPA) or *S*. Typhi (ST) against STY0796 (A), STY1086 (B), STY4190 (C) and STY3208 (D) antigens over the course of three months. Differences between time points were assessed using Kruskal-Wallis test followed by pairwise comparisons using Wilcoxon signed-rank tests. P-values were adjusted using the Bonferroni multiple testing correction method. *p<0.05, **p<0.01, ***p<0.001, ****p<0.0001.


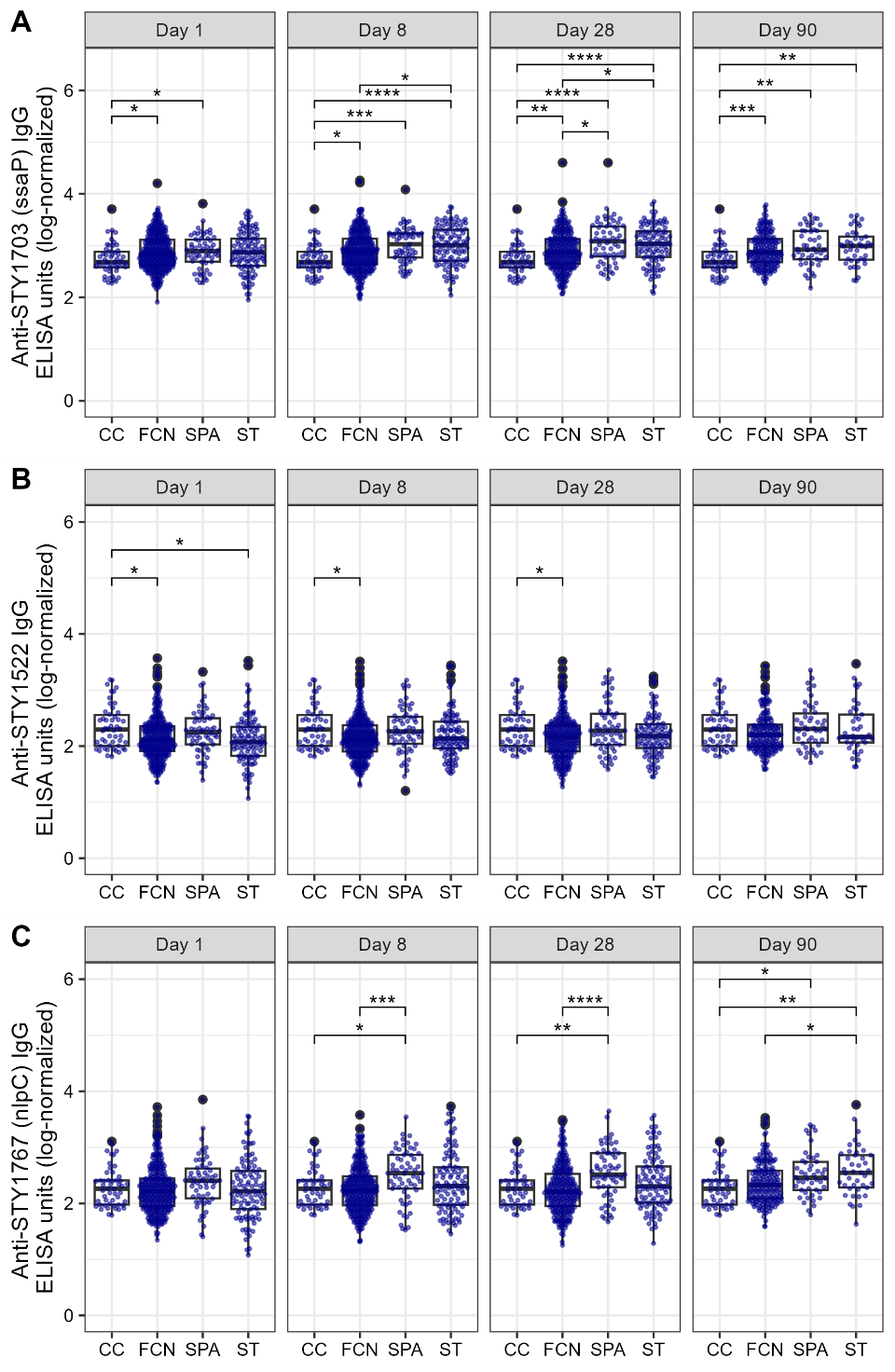


Figure S12. The distribution of serum IgG titers in a Nepali cohort of febrile patients and controls.

Boxplots showing IgG levels in plasma from controls (Ctrl), culture-negative patients (FCN), *S*. Paratyphi A (SPA) or *S*. Typhi (ST) against STY1703 (A), STY1522 (B) and STY1767 (C) antigens over the course of three months. Differences between time points were assessed using Kruskal-Wallis test followed by pairwise comparisons using Wilcoxon signed-rank tests. P-values were adjusted using the Bonferroni multiple testing correction method. *p<0.05, **p<0.01, ***p<0.001, ****p<0.0001.

# References

1. R Core Team. R: a language and environment for statistical computing [Internet]. Vienna, Austria: R Foundation for Statistical Computing, Vienna, Austria; 2019. Available from: https://www.r-project.org/

2. Wickham H. tidyverse: easily install and load the “Tidyverse” [Internet]. 2017. Available from: https://cran.r-project.org/package=tidyverse

3. Sjoberg DD, Whiting K, Curry M, Lavery JA, Larmarange J. Reproducible Summary Tables with the gtsummary Package. R J. Technische Universitaet Wien; **2021**; 13(1):570–580.

4. Kassambara A. ggpubr: “ggplot2” Based Publication Ready Plots [Internet]. 2020. Available from: https://cran.r-project.org/package=ggpubr

5. Kassambara A. rstatix: Pipe-Friendly Framework for Basic Statistical Tests [Internet]. 2020. Available from: https://cran.r-project.org/package=rstatix

6. Wilke CO. cowplot: Streamlined Plot Theme and Plot Annotations for “ggplot2” [Internet]. 2020. Available from: https://cran.r-project.org/package=cowplot

7. Clarke E, Sherrill-Mix S. ggbeeswarm: Categorical Scatter (Violin Point) Plots [Internet]. 2017. Available from: https://cran.r-project.org/package=ggbeeswarm

8. Kassambara A. ggcorrplot: Visualization of a Correlation Matrix using “ggplot2” [Internet]. 2019. Available from: https://cran.r-project.org/package=ggcorrplot

9. Barret S, Di C, JLarmarange J, et al. GGally: Extension to ’ggplot2 [Internet]. 2012. Available from: https://cran.r-project.org/package=GGally

10. Firke S. janitor: Simple Tools for Examining and Cleaning Dirty Data [Internet]. 2021. Available from: https://cran.r-project.org/package=janitor
